# The Long Noncoding RNA MALAT1 Induces Tolerogenic Dendritic Cells and Regulatory T Cells *via* miR155/Dendritic Cell-Specific Intercellular Adhesion Molecule-3 Grabbing Nonintegrin/IL10 Axis

**DOI:** 10.3389/fimmu.2018.01847

**Published:** 2018-08-13

**Authors:** Jian Wu, Hanlu Zhang, Yang Zheng, Xiangyuan Jin, Mingyang Liu, Shuang Li, Qi Zhao, Xianglan Liu, Yongshun Wang, Ming Shi, Shengnan Zhang, Jinwei Tian, Yong Sun, Maomao Zhang, Bo Yu

**Affiliations:** ^1^Department of Cardiology, The Second Affiliated Hospital of Harbin Medical University, Harbin, China; ^2^The Key Laboratory of Myocardial Ischemia, Harbin Medical University, Ministry of Education, Harbin, China; ^3^Department of Thoracic Surgery, The Third Affiliated Hospital of Harbin Medical University, Harbin, China; ^4^School of Biomedical Sciences, The University of Hong Kong, Pokfulam, China; ^5^School of Life Science and Technology, Harbin Institute of Technology, Harbin, China

**Keywords:** MALAT1 long noncoding RNA, tolerogenic dendritic cell, immune tolerance, dendritic cell-specific intercellular adhesion molecule-3 grabbing nonintegrin, miR155, IL10

## Abstract

By shaping T cell immunity, tolerogenic dendritic cells (tDCs) play critical roles in the induction of immune tolerance after transplantation. However, the role of long noncoding RNAs (lncRNAs) in the function and immune tolerance of dendritic cells (DCs) is largely unknown. Here, we found that the lncRNA MALAT1 is upregulated in the infiltrating cells of tolerized mice with cardiac allografts and activated DCs. Functionally, MALAT1 overexpression favored a switch in DCs toward a tolerant phenotype. Mechanistically, ectopic MALAT1 promoted dendritic cell-specific intercellular adhesion molecule-3 grabbing nonintegrin (DC-SIGN) expression by functioning as an miR155 sponge, which is essential for the tolerogenic maintenance of DCs and the DC-SIGN-positive subset with more potent tolerogenic ability. The adoptive transfer of MALAT1-overexpressing DCs promoted cardiac allograft survival and protected from the development of experimental autoimmune myocarditis, accompanied with increasing antigen-specific regulatory T cells. Therefore, overexpressed MALAT1 induces tDCs and immune tolerance in heart transplantation and autoimmune disease by the miRNA-155/DC-SIGH/IL10 axis. This study highlights that the lncRNA MALAT1 is a novel tolerance regulator in immunity that has important implications in settings in which tDCs are preferred.

## Highlights

1. MALAT1 overexpression favors a switch of LPS-stimulated DCs toward a tolerant phenotype.
2. miR155/DC-SIGN/IL10 is essential for the tolerogenic function mediated by MALAT1.
3. DC-SIGN^+^ subsets induced by ectopic MALAT1 exert more potent immunotolerant ability.
4. MALAT1-overexpressing DCs mediate cardiac allograft tolerance and protect from EAM in mice.

## Introduction

Dendritic cells (DCs), as the most potent APCs, control the fate of the immune response and play critical roles in transplant immunity, the autoimmune response, cancer immunity, and infective immunity (1, 2). DCs present antigens and interact with T cells to shape T cell differentiation and function. The state of DCs, including their maturation, function, and cytokine microenvironment, determines the tendency of DC–T cell interactions toward immunity or tolerance (1, 2). Tolerogenic dendritic cells (tDCs), which have a tolerogenic phenotype, induce antigen-specific tolerance through the induction of T cell anergy and Treg expansion or an anti-inflammatory cytokine environment (3, 4). Thus, tDCs are attractive targets for therapeutic approaches aiming to establish immune tolerance after transplantation or in autoimmune diseases (5–8). Elucidating the tolerance regulators involved in the functional state of DCs would facilitate the development of new interventional strategies for inducing allograft immune tolerance.

Dendritic cell-specific intercellular adhesion molecule-3 grabbing nonintegrin (DC-SIGN) is an innate immune receptor mainly expressed by DCs and macrophages. This receptor is involved in many aspects of DCs, including pathogen recognition and antigen presenting, and is thus considered a functional hallmark of DCs. DC-SIGN mediates the functions of DCs and macrophages in presenting and shaping T cell immunity (9–13). Due to its immune regulatory functions, DC-SIGN is also involved in immunosuppressive maintenance after transplantation and during tumor growth and pathogenic infection (14, 15). Most importantly, DC-SIGN actively contributes to the induction of allograft immune tolerance after transplantation (15). It has been reported that DC-SIGN is indirectly targeted by miR155, a prominent regulator of DC function and allograft immunity (16–20), *via* direct inhibition of PU.1 (21).

Long noncoding RNAs (lncRNAs) exert their pathophysiologic effects systemically and have emerged as critical regulators of the immune response (22–24). In addition, lncRNAs have been linked to transplant rejection and DC differentiation (25, 26), but the role of lncRNAs in the functional modulation of DCs and immune tolerance induction is largely unknown. The MALAT1 lncRNA was initially discovered in tumors and was recently reported to be involved in the innate immune response (27, 28). In this study, we first identified the lncRNA MALAT1 in tolerized cardiac allografts and further elucidated the contribution of MALAT1 to the tolerogenic function of DCs and immune tolerance induction in heart transplantation and autoimmune disease.

## Materials and Methods

### Animals

Adult male C57BL/6 and BALB/c mice (4–6 weeks old, weighing 15–20 g) were purchased from the Shanghai Lab Animal Research Center (China). All experimental protocols were approved by the Institutional Animal Care and Use Committee at Harbin Medical University. This study was conducted in accordance with the Guide for the Care and Use of Laboratory Animals (Institute of Laboratory Animal Resources/National Institutes of Health, Bethesda, MD, USA).

### Heart Transplantation and Cell Transfer

After general anesthesia, the BALB/c recipients were transfused with phosphate-buffered saline (PBS) or conditioned DCs by intravenous injection into the penile vein. At 24 h after transfusion, the BALB/c recipients underwent fully vascularized heterotopic heart transplantation of a C57BL/6 murine heart using microsurgical techniques (29). After cardiac transplantation, several recipient mice were orally administered 1 mg/kg tacrolimus (positive control). For tolerance induction, several recipient mice were treated with anti-CD40L mAb (250 µg, BioXcell) at 0, 2, and 4 days post-transplantation (15). Post-operatively, graft survival was assessed daily for allograft cardiac contraction by palpation. Complete cessation of the heartbeat and histologic examination of the graft were used to define allograft rejection.

### Experimental Autoimmune Myocarditis (EAM) Induction and DC Transfusion

BALB/c mice were immunized with α-myosin H-chain peptide (200 µg; MyHC-α 614–629 [Ac-S LKLM ATLFSTYAS AD-OH]; Ontores Biotechnologies Co., Ltd., Zhejiang, China) emulsified 1:1 in PBS and complete Freund’s adjuvant (Sigma-Aldrich Corp., St. Louis, MO, USA) on days 0 and 7. For the *in vivo* experiments, BALB/c mice were transfused with PBS, LPS-treated DC, or MALAT1-overexpressing DCs by intravenous injection into the penile vein at days 1, 4, and 7 post-immunization. Hearts were collected after 21 and 42 days of immunization.

### Histologic Analyses of the Cardiac Allografts

Allografts from the recipients were harvested on day 7 after transplantation. Half of the allografts were embedded in paraffin for hematoxylin and eosin (H&E) staining. In addition, paraffin-embedded sections were stained for Foxp3 (WanleiBIO, China). Images were captured using an Olympus BX4 l microscope. H&E staining was assessed by grading from 0 (none) to 3 (severe), according to the 2005 classification of the International Society for Heart and Lung Transplantation for Acute Cellular Rejection. Scoring was performed *via* light microscopy in a blinded fashion.

### Generation of Bone Marrow-Derived DCs (BMDCs)

Bone marrow-derived DCs were generated from the BM cells of male BALB/c mice. These cells were cultured with GM-CSF (20 ng/ml) and IL4 (10 ng/ml) in RPMI 1640 medium (HyClone) supplemented with 10% FBS (Sciencell) (30). The culture medium was replenished every 2 days. The DCs were conditioned with LPS (200 ng/ml, Sigma-Aldrich, St. Louis, MO, USA) for 12 h on day 6 unless otherwise indicated.

### Transfection and Treatment of DCs

Dendritic cells were treated with TNFα (25 ng/ml, PharMingen), TLR3 ligands (polyinosinic-polycytidylic acid, 2 µg/ml, Sigma-Aldrich), and TLR5 ligand (flagellin, 0.1 µg/ml, InvivoGen). For MALAT1 upregulation, cDNA encoding lncRNA MALAT1 (position: 3201–5600, length 2,400 bp) was PCR-amplified and subcloned into the pcDNA3.1 vector. Interfering RNAs (siRNA) that specifically target mouse MALAT1 were purchased from RiboBio Smart Silencer™. The mouse miR-155 mimic and inhibitor were purchased from GenePharma (Shanghai, China). DCs were transfected with the MALAT1 pcDNA3.1 vector (pMALAT1, 2.5 µg/ml), control vector (Vector, 0.625 µg/ml), MALAT1 siRNA (siMALAT1, 100 nM), or siRNA control (siNC, 25 nM) using Lipofectamine 2000 (Invitrogen) for 6 h on day 6 before LPS stimulation, according to the manufacturer’s protocol. To inhibit NF-κB activity in BMDCs, at day 6, PDTC (50 µM, 30 min, Abcam) or SC-514 (100 mM; Sigma-Aldrich) was used before the LPS treatment. In several experiments, DCs were conditioned with siRNA targeting DC-SIGN (25 nM; GenePharma, China).

### FISH

Briefly, DCs were fixed in 4% paraformaldehyde and washed. The prehybridization solution, hybridization solution, and MALAT1 probe were purchased in a RiboBio™ Fluorescent *In Situ* Hybridization Kit (RiboBio, China). The cells were prehybridized with the prehybridization solution and then incubated with a MALAT1 probe in hybridization solution at 37°C overnight. After 24 h, the cells were washed with 4× SSC, 2× SSC, and 1× SSC and then counterstained with DAPI. Images were captured using a fluorescence microscope (DM 4000B, Leica, Germany). The harvested allograft samples were immediately frozen in liquid nitrogen and then cut into 5-μm-thick sections and adhered to slides. After washing and fixing, the tissue sections were used for FISH assays. Similarly, the tissue sections of allografts were also prehybridized and incubated with the MALAT1 probe at 37°C overnight. The sections were washed and counterstained with DAPI. Images were captured using a confocal laser-scanning microscope (FluoView v5.0FV300; Olympus, Tokyo, Japan).

### Graft-Infiltrating Lymphocyte Isolation

The cardiac allografts were harvested from recipient mice on day 7 post-transplantation, and graft-infiltrating lymphocytes were isolated from cardiac grafts using published protocols. Briefly, grafts were minced and incubated with 2 mg/ml collagenase for 2 h. The infiltrating cells were purified using Percoll and then placed in RLT lysis buffer (Qiagen, Hilden, Germany).

### Microarray Analysis

Long noncoding RNA microarrays were used to profile lncRNA and mRNA expression from three tolerized cardiac allografts and three rejected allografts samples by OEBiotech (Shanghai, China). The cRNAs were hybridized onto an Agilent Mouse Gene Expression Array (Agilent Technologies, CA, USA). When the RNA expression level changed by at least twofold with a *P*-value <0.05, the lncRNA or mRNA expression was considered significantly different. The differentially expressed lncRNAs between the two groups were identified *via* heatmaps and volcano plot filtering.

### Flow Cytometry

Bone marrow-derived DC flow cytometry was performed with the following antibodies from BD Biosciences: anti-CD11c FITC, anti-CD80 PE, anti-CD86 PE, anti-MHC II PE, and anti-DC-SIGN PE. T cell flow cytometry was performed with the following antibodies from BD Biosciences: anti-CD4 FITC, anti-CD25 APC, anti-Foxp3 PE, anti-IFN PE, and anti-IL17A PE. Intracellular staining was performed using the Intracellular Fixation & Permeabilization Buffer Set (eBioscience). For IFN and IL17A staining, cells were first treated with 500 ng/ml ionomycin (Sigma) and 50 ng/ml phorbol myristate acetate (Sigma) in the presence of 1 µg/ml GolgiPlug (Sigma) for 5 h at 37°C. Flow cytometry data were acquired on a FACSCanto II system (BD Biosciences), and the data were analyzed using Flow Jo software.

### Mixed Lymphocyte Reaction (MLR) Assay

Dendritic cells, as stimulators for MLRs, were isolated from BALB/c mice. Allogenic splenic T cells (from C57BL/6 mice) were cultured for 3 days in cell plates in the presence of 10 µg/ml mitomycin C-pretreated DCs at a DC:T cell ratio of 1:10. Proliferation was assessed using a BrdU-ELISA according to the manufacturer’s instructions (Chemicon International, Temecula, CA, USA). The immunosuppressive function of isolated Tregs was also assessed by BrdU-ELISA. Tregs (CD4^+^CD25^+^) were added into MLR cocultures of mitomycin C-treated stimulators (splenic cells or DCs) and allogeneic effector T cells in a Treg:T cell:stimulator ratio of 10:10:1. Effector T cell proliferation was assessed by BrdU-ELISA.

### Cell Sorting by MACS

In MLR culture mixtures, Tregs (CD4^+^CD25^+^) were separated by MACS. First, CD4^+^ T cells were enriched by negative selection with microbeads (Miltenyi Biotec). Then, the isolated CD4^+^ T cell population was positively selected with anti-CD25 mouse microbeads into CD4^+^CD25^+^ and CD4^+^CD25^−^ T cell fractions (Miltenyi Biotec). Flow cytometry analysis was used to confirm a Treg isolation purity (CD4^+^CD25^+^) between 80 and 90%. The DC-SIGN^+^ DC population was positively selected with anti-PE magnetic beads (Miltenyi Biotec) after staining with PE-DC-SIGN Ab (eBioscience). The purity of the DC-SIGN^+^ DC population was also assessed by FACS and was typically between 80–90%.

### RNA Isolation and Quantitative Real-Time Reverse Transcription PCR (qRT-PCR)

The total RNA was isolated from DCs with TRIzol reagent (Invitrogen) according to the manufacturer’s protocol. Nuclear and cytoplasmic extracts were prepared using cytoplasmic and nuclear RNA purification kits (Norgen Biotek, Canada) according to the manufacturer’s instructions, and this was followed by RNA isolation. lncRNA MALAT1, DC-SIGN, and IL10 cDNA transcripts were amplified using a Transcriptor First Strand cDNA Synthesis Kit (Roche). The PCR mixture was prepared using Fast Start Universal SYBR Green Master Mix (Roche). For miR-155, RT-PCR was performed using TaqMan miRNA assay kits following the manufacturer’s instructions (Genepharma, China). Each sample was measured in triplicate. The results were normalized to β-actin or U6 and analyzed using the 2^−ΔΔCt^ method. The primer sequences are found in Table S1 in Supplementary Material.

### ELISA

To measure the secretion of cytokines by DCs, supernatants were collected from the cultures after transfection and LPS stimulation. The levels of IL12, IL6, IFNγ, TGFβ, and IL10 (BD Biosciences, CA, USA) were determined by ELISA according to the manufacturer’s instructions. In several experiments, the cytokine levels in the plasma of recipient mice 7 days after transplantation were also measured. All assays were performed in triplicate.

### Dual-Luciferase Assay

Mouse MALAT1-WT and a mutant derivative devoid of any miR-155 binding site (MALAT1-mut) were cloned downstream of the coding region of the luciferase gene. DCs were infected with or without miR-155 mimics and then, respectively, transfected with luciferase constructs of WT1, WT2, WT3, Mut1, Mut2, or Mut3 using Lipofectamine 2000 in 293T cells. Luciferase assays were performed using a luciferase assay kit (Promega, USA) according to the manufacturer’s instructions.

### Western Blotting

Western blotting was used to assess the expression of NF-κB p65, SOCS1, PU-1, DC-SIGN, STAT3, and pSTAT3 in the DCs. Briefly, DC lysates were obtained and then blotted to determine NF-κB, PU-1, DC-SIGN, STAT3, pSTAT, and β-actin expression using anti-NF-κB, anti-PU-1, anti-DC-SIGN (CD209a), anti-STAT3, anti-pSTAT3, and anti-β actin antibodies (Abcam, USA). Protein levels were quantified using scanning densitometry (GS-710 imaging). Data were obtained in triplicate from independent experiments.

### ChIP

ChIP assays were performed using a ChIP Kit according to the manufacturer’s instructions (Pierce, USA). NF-κB p65 antibodies were obtained from Abcam. Quantification of immunoprecipitated DNA was performed using qPCR. The ChIP primer sequences are listed in Table S1 in Supplementary Material. ChIP data were determined as a percentage relative to the input DNA using Equation 2: [Input Ct − Target Ct] × 0.1 × 100.

### RNA Immunoprecipitation (RIP)

RNA immunoprecipitation was performed using a Magna RIP RNA-Binding Protein Immunoprecipitation Kit (Millipore, USA) according to the manufacturer’s instructions. Anti-NF-κB p65 (Abcam, USA) or anti-Ago2 antibodies (Bioworld, USA) were used to obtain the coprecipitated RNAs in the RIP assay. Then, the obtained RNAs were analyzed by qPCR with the primers listed in Table S1 in Supplementary Material. The total RNA (input control) and the isotype control were used to confirm the binding specificity of RNAs with NF-κB p65 or Ago.

### Statistical Analyses

The data are expressed as the mean ± SD. Cardiac allograft survival in each group of mice was compared using the Mann–Whitney *U* test. Other data were compared using analysis of variance with the Ryan method. A value of *P* < 0.05 was considered the threshold for statistical significance. The data were analyzed using GraphPad Prism software.

## Result

### MALAT1 Is Upregulated in LPS-Stimulated DCs Transcriptionally Activated by NF-κB

To determine whether lncRNA was involved in immune tolerance induction after transplantation, lncRNA microarray analysis was performed with tolerized (induced by anti-CD40L mAb treatment) and rejected cardiac allografts in mice. A significant aberrant expression profiling between the two groups was found, and 50 differentially expressed lncRNAs are shown in Figure S1A in Supplementary Material. Among those altered lncRNAs, we identified the lncRNA MALAT1 as enriched and upregulated in tolerized cardiac allografts (Figure S1B in Supplementary Material). Moreover, MALAT1 was also significantly upregulated in the graft-infiltrating cells of tolerized allografts (Figure S1C in Supplementary Material). To reveal the location of possible target infiltrating cells for MALAT1, a FISH assay for MALAT1 was performed, and it was found that MALAT1 was co-localized with CD11c^+^ cells in tolerized allograft cardiac tissue (Figure S1D in Supplementary Material). These data indicated that MALAT1 might be involved in immune tolerance regulation *via* acting on the DCs function. Therefore, we further explored the potential effect of MALAT1 on DCs in an *in vitro* study.

**Figure 1:**
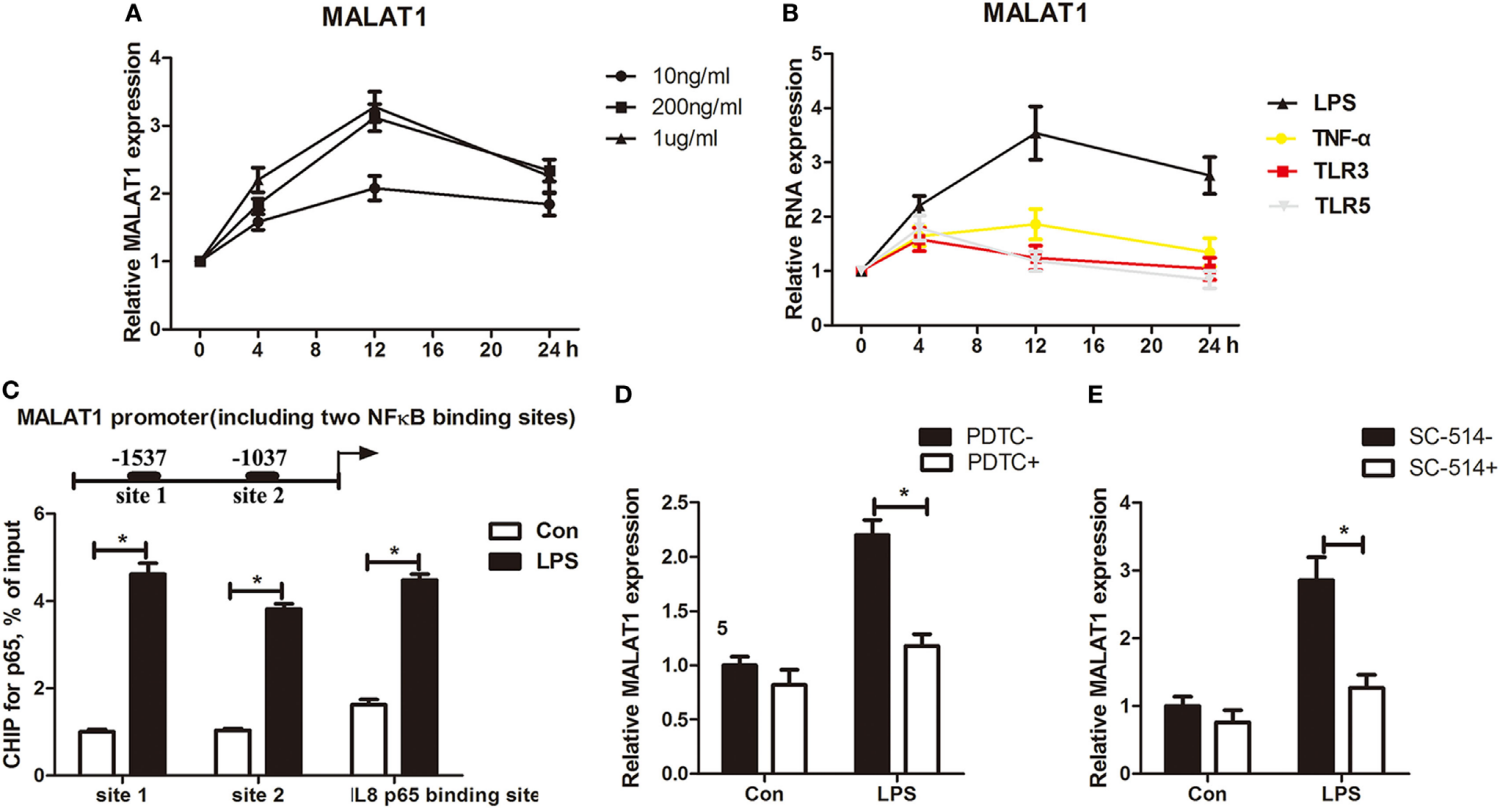
MALAT1-activated LPS-stimulated dendritic cells (DCs) transcriptionally regulated by NF-κB. **(A)** Quantitative real-time reverse transcription PCR (qRT-PCR) was performed to detect MALAT1 expression in DCs stimulated with LPS (10, 200, or 1 µg/ml) at different time points (0, 4, 12, and 24 h). MALAT1 expression was upregulated, reaching peak expression 12 h after LPS stimulation. **(B)** qRT-PCR analysis of the expression of MALAT1 in DCs stimulated with LPS (200 ng/ml), TNFα (25 ng/ml), the TLR3 agonist lipoteichoic acid (1 mg/ml), or the TLR5 agonist polyinosinic-polycytidylic acid (2 µg/ml) for 24 h (*n* = 5). MALAT1 levels were upregulated after the application of TNFα, TLR3 ligand, and TLR5 ligand. **(C)** A ChIP assay was performed with extracts from DCs treated with LPS (200 ng/ml, 12 h) and an antibody against NF-κB p65. The p65 binding site in the promoter region of the IL-8 gene acted as a positive control. The immunoprecipitated DNA was amplified using primers specific for the two predicted p65 binding sites of the MALAT1 promoter. Binding of p65 to both the binding sites at −1537 and at −1037 of the MALAT1 gene following LPS stimulation was increased. **(D)** qRT-PCR was performed to detect MALAT1 expression in DCs pretreated with or without PDTC (50 µM, 30 min) before LPS stimulation (*n* = 5). PDTC significantly blocked the LPS-induced increase in MALAT1 levels in LPS-stimulated DCs. **(E)** MALAT1 expression detected by qRT-PCR in DCs pretreated with or without another inhibitor of IKK2, SC-514 (100 mM). SC-514 significantly blocked the LPS-induced increase in MALAT1 levels in LPS-stimulated DCs. All values of long noncoding RNA (lncRNA) expression levels were normalized to β-actin. The data are presented as the mean ± SD from at least three independent experiments. *P < 0.05.

To investigate the roles of MALAT1 in DCs, we first analyzed the expression pattern of MALAT1 during the process of DC maturation in response to LPS for 4, 12, and 24 h. As assessed by qRT-PCR, MALAT1 expression was upregulated, reaching peak expression 12 h after different doses of stimulatory LPS were applied (Figure 1A). MALAT1 levels were also upregulated after the application of other stimuli (TNFα, TLR3 ligand, and TLR5 ligand); however, the upregulation of MALAT1 induced by other stimuli was significantly lower than that induced by LPS stimulation, especially at 12 h (Figure 1B). Thus, in subsequent experiments, DCs were treated with 200 ng/ml LPS for 12 h unless otherwise indicated. As NF-κB is a critical mediator and transcription factor involved in TLR stimulation, we next investigated whether NF-κB is necessary for MALAT1 transcription. We predicted two potential binding sites of NF-κB p65 in the MALAT1 promoter by bioinformatic analysis with ChIPBase (http://rna.sysu.edu.cn/chipbase/). Increased binding of p65 to the both binding sites at −1537 and −1037 of the MALAT1 gene following LPS stimulation was demonstrated by ChIP analysis using specific primers for each putative binding site, with the difference at site 1 being more significant (Figure 1C). The p65 binding site in the promoter region of the IL-8 gene acted as a positive control. To further test the potential transactivation of the MALAT1 gene by the p65 subunit, we used PDTC, an inhibitor of NF-κB, to knock down NF-κB expression in LPS-stimulated DCs for 4 h. We found that PDTC significantly blocked the LPS-induced increase in MALAT1 levels in LPS-stimulated DCs (Figure 1D). Likewise, we tested another IKK2 inhibitor, SC-514, which inhibits p65-associated transcriptional activation of the NF-κB pathway, and we obtained a similar result (Figure 1E). These findings indicated that MALAT1 was upregulated in LPS-stimulated DCs and the transcription of MALAT1 is partially NF-κB dependent.

### Ectopic MALAT1 Favors tDCs by Inducing T Cell Hyporesponsiveness and Treg Expansion

**Figure 2:**
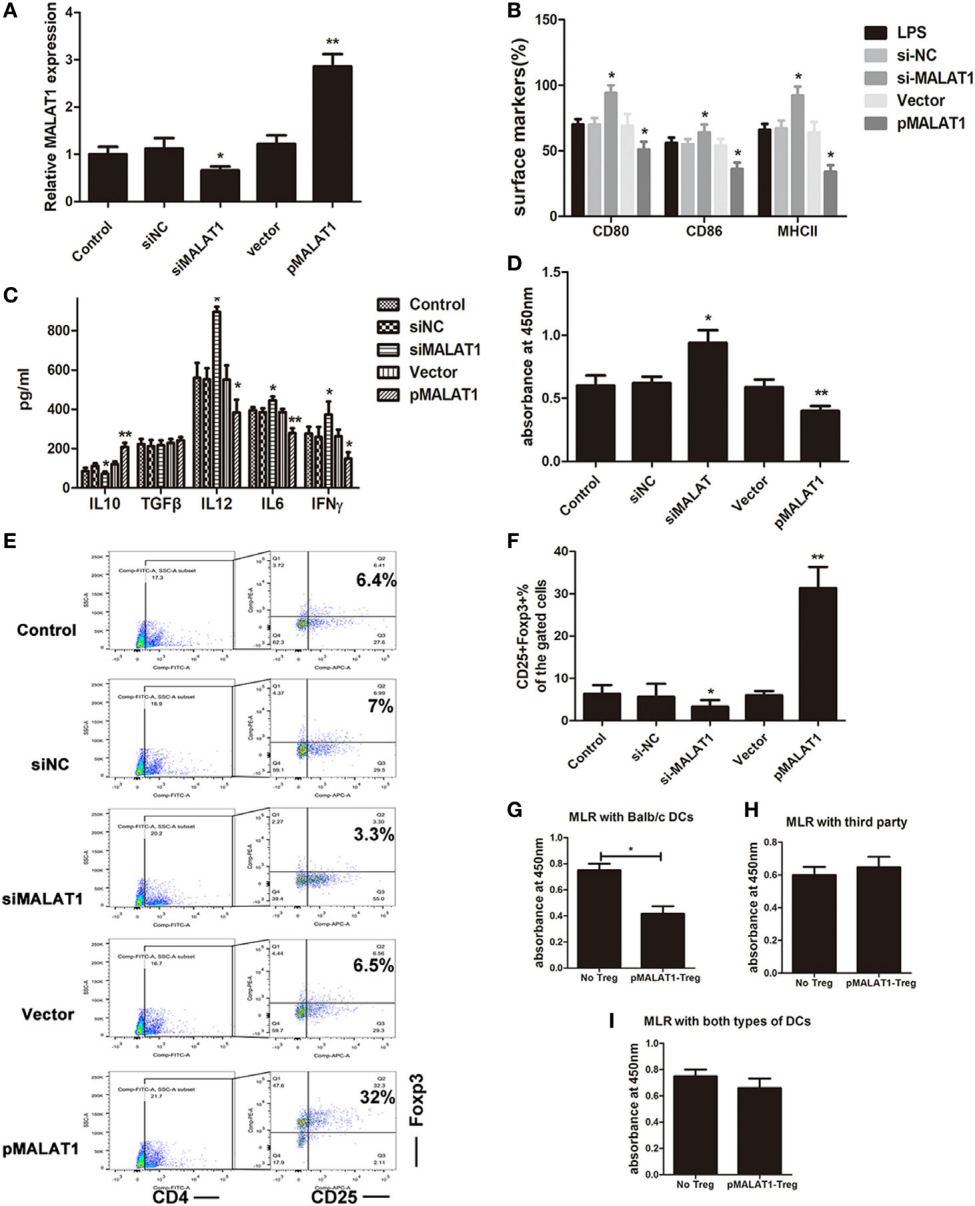
Ectopic MALAT1 favors tolerogenic dendritic cells (tDCs) by inducing T cell hyporesponsiveness and Tregs expansion. Dendritic cells (DCs) were transfected with a MALAT1 pcDNA3.1 vector (pMALAT1, 2.5 µg/ml), a control vector (Vector, 0.625 µg/ml), MALAT1 siRNA (siMALAT1, 100 nM), or siRNA control (siNC, 25 nM) for 6 h before LPS treatment. **(A)** The expression of MALAT1 was confirmed by quantitative real-time reverse transcription PCR in DCs receiving the different treatments. Expression of MALAT1 was effectively upregulated by pMALAT1, whereas MALAT1 siRNA suppressed MALAT1 expression in DCs. **(B)** Expression of the costimulatory markers CD80, CD86, and MHCII was analyzed by flow cytometry and is shown as the percentage of CD11c^+^ cells. MALAT1-suppressed DCs displayed increases in CD80, CD86, and MHCII expression levels compared with those of control DCs treated with LPS. **(C)** The cytokine secretion levels of IL10, TGFβ, IL12, IFNγ, and IL6 in the DC supernatants were analyzed by ELISA. The induced expression of MALAT1 in LPS-stimulated DCs resulted in reduced levels of IL6, IL12, and IFNγ, whereas levels of IL10 were significantly increased, but no significant effect was observed on TGF-β. **(D)** DC-triggered T cell proliferation was evaluated by BrdU-ELISA. For this, DCs were treated with mitomycin C (10 mg/ml, 2 h) and cocultured with allogeneic T cells for 48 h at a DC:T cell ratio of 1:20. T cells cocultured with MALAT1-overexpressing DCs exhibited reduced proliferative ability compared with those cocultured with LPS-DCs. **(E,F)** In the cocultured T cells, the numbers of Tregs (CD4^+^, CD25^+^, and Foxp3^+^) cells were assessed by flow cytometry. Boxes depict gates and numbers correspond to the percentage of cells in each gate. The data are shown with a representative flow cytometry **(E)** and the percentages **(F)**. The number of Tregs was significantly increased when T cells were cocultured with MALAT1-overexpressing DCs compared with LPS-DCs. **(G–I)** Tregs (CD4^+^CD25^+^) isolated from T cells cocultured with pMALAT1-conditioned DCs by MACS were added into coculture with DCs (from BALB/c, third-party mice or both types ratio of 1:1) and T cells, with a Treg:T cell:DC ratio of 10:10:1. Then, the suppressive ability of the Tregs was assessed by T cell proliferation assays using BrdU-ELISA. Tregs derived from MALAT1-overexpressing DC (from BALB/c mice) cocultures suppressed T cell proliferation in the presence of BALB/c DCs as stimulators but not in the presence of DCs from third-party mice, whereas there was no significant difference in T cell proliferation between both types of LPS-induced DCs. The data are presented as the mean ± SD from at least three independent experiments. *P < 0.05; **P < 0.01. pMALAT1-Treg, Tregs induced by pMALAT1-conditioned DCs in MLRs; No-Treg, without Tregs.

To clarify the potential role of LPS-induced MALAT1 in shaping the DC phenotype, we then constructed lentiviruses containing siRNAs targeting MALAT1 and plasmids to overexpress MALAT1 (pMALAT1), transduced them into LPS-stimulated DCs for 6 h, and found that the expression of MALAT1 could be effectively upregulated by pMALAT1, whereas MALAT1 siRNA suppressed MALAT1 expression in DCs (Figure 2A). Then, we investigated the effects of gain- or loss-of-function MALAT1 on the phenotypic and functional characteristics of the DCs. MALAT1-suppressed DCs displayed increases in CD80, CD86, and MHCII expression levels compared with control DCs treated with LPS (Figure 2B). By contrast, MALAT1-overexpressing DCs expressed lower levels of CD80, CD86, and MHCII than did LPS-DCs, which reflected that MALAT1 depressed LPS-induced DC maturation.

Accumulating evidence shows that DCs secrete cytokines in response to various types of stimuli to rebalance the microenvironment. In particular, increased IL-10 (immunosuppressive cytokine) is the dominant change mediating immune tolerance. Thus, we analyzed cytokine production in the supernatant from DCs pretreated with pMALAT1 or siMALAT1 by ELISA. The induced expression of MALAT1 in LPS-stimulated DCs resulted in reduced levels of the pro-inflammatory cytokines IL6, IL12, and IFNγ, whereas the levels of the anti-inflammatory cytokine IL10 were significantly increased, but no significant effect on TGF-β was observed (Figure 2C). Therefore, MALAT1 overexpression shaped LPS-stimulated DCs into a tolerogenic phenotype with low costimulatory markers and high IL10 secretion.

Tolerogenic dendritic cells have been reported to induce immune tolerance by shaping T cell activity and generation of immunosuppressive Tregs. Accordingly, DC-primed T cell proliferation was evaluated in T cells cocultured with BMDCs in a MLR assay. T cells cocultured with MALAT1-overexpressing DCs exhibited reduced proliferative ability compared with those cocultured with LPS-DCs (Figure 2D). Under the same conditions, the number of Tregs was significantly increased when T cells were cocultured with MALAT1-overexpressing DCs compared with LPS-DCs (Figure 2E). The differences in the percentage of Tregs were documented quantitatively (Figure 2F). Boxes depict gates and numbers correspond to percentage of cells in each gate (Figure S2 in Supplementary Material). To further investigate the mediated immunosuppression of Tregs in MALAT-conditioned DCs, we separated CD4^+^CD25^+^ Tregs from the culture mixture of MALAT1-overexpressing DCs (from BALB/c mice) and T cells by MACS, and then we cocultured purified CD4^+^CD25^+^ Tregs with T cells and LPS-induced DCs (from BALB/c, third-party mice or both types) at a ratio of 10:10:1 and assessed T cell proliferation assays by BrdU-ELISA. Significantly, Tregs derived from MALAT1-overexpressing DC (from BALB/c mice) cocultures exhibited suppressed T cell proliferation in the presence of BALB/c DCs as stimulators but not in the presence of DCs from third-party mice (Figures 2G,H), whereas there was no significant difference in T cell proliferation between both types of LPS-induced DCs (Figure 2I). Overall, these results confirm that DCs with MALAT1 overexpression are tolerogenic, thereby impairing the activation of effector T cell responses and inducing Tregs with antigen-specific inhibitory effects.

### DC-SIGN Is Essential for the Maintenance of MALAT1-Conditioned DC Tolerogenic Functions

**Figure 3:**
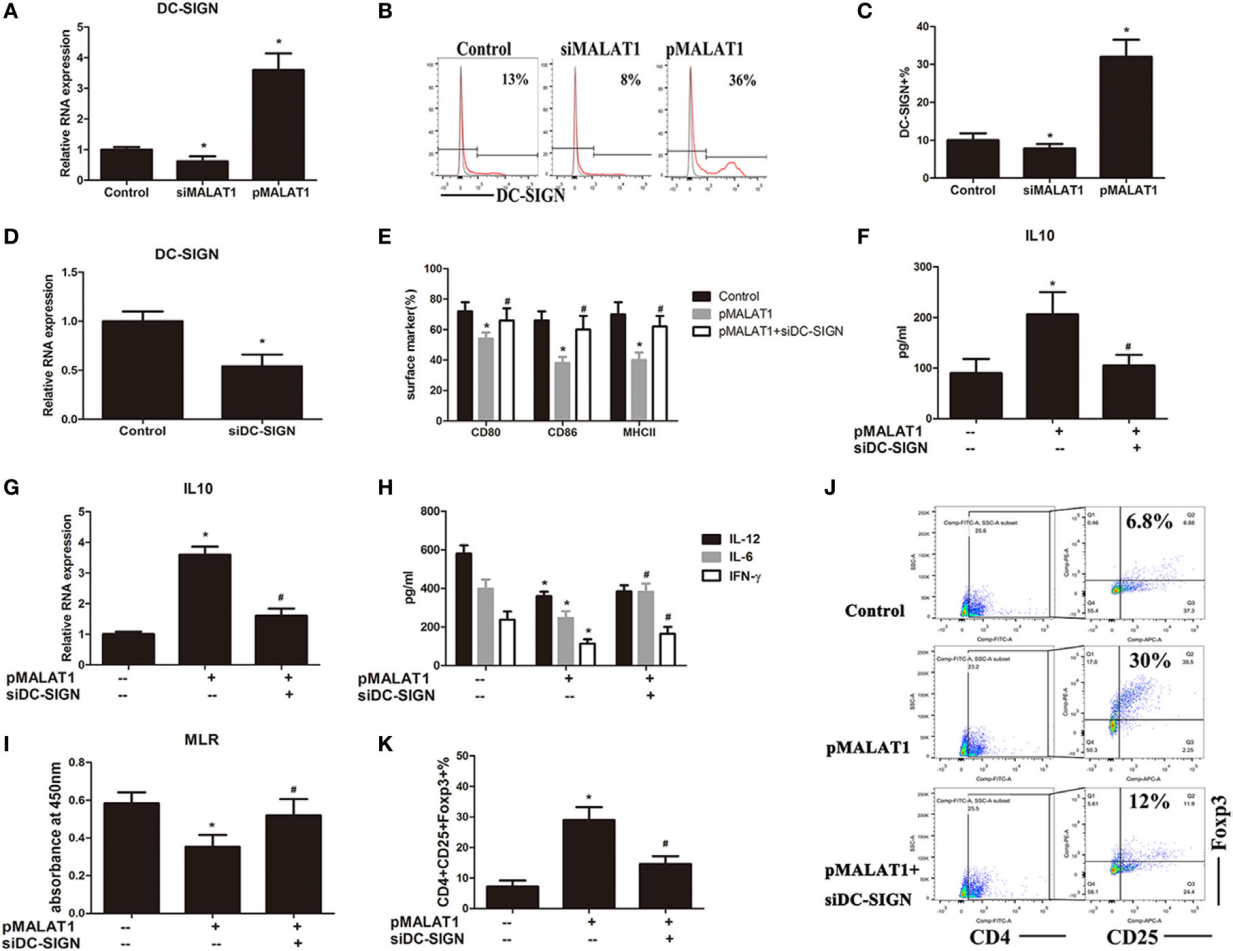
Dendritic cell-specific intercellular adhesion molecule-3 grabbing nonintegrin (DC-SIGN) is essential for the maintenance of MALAT1-conditioned dendritic cell (DC) tolerogenic functions. **(A–C)** DCs were transfected with siMALAT1 or pMALAT1 for 6 h before LPS stimulation. **(A)** The expression of DC-SIGN was detected by quantitative real-time reverse transcription PCR (qRT-PCR). The mRNA expression of DC-SIGN was significantly increased in DCs with overexpressed MALAT1 but decreased in those with MALAT1 shRNA compared with control DCs. **(B)** The level of DC-SIGN was assessed by flow cytometry and shown as percentages. **(C)** The DC-SIGN expression in DCs was significantly upregulated by MALAT1 overexpression, while MALAT1 downregulation counteracted this effect. **(D)** The expression of DC-SIGN was detected by qRT-PCR in DCs transfected with siDC-SIGN or the control (25 nM, 6 h). DC-SIGN-targeted shRNA transfection obviously downregulated the expression of DC-SIGN in DCs. **(E–K)** DCs were pre-incubated with or without shDC-SIGN for 6 h and subsequently cultured with the pMALAT1 vector before LPS stimulation. **(E)** Expression of the costimulatory markers CD80, CD86, and MHCII was assessed by flow cytometry. In DCs pretreated with the pMALAT1 vector plus DC-SIGN shRNA, the costimulatory molecules were significant increased compared with DCs only treated with the pMALAT1 vector, with no obvious difference compared to control non-treated DCs. **(F)** The cytokine secretion levels of IL10 in the DC supernatants were analyzed by ELISA. **(G)** The mRNA expression of IL10 in the DCs was analyzed by qRT-PCR. DC-SIGN knockdown by DC-SIGN shRNA impaired the MALAT1-induced upregulation of IL10 protein and mRNA expression in DCs. **(H)** The cytokine secretion levels of IL6, IL12, and IFNγ in the DC supernatants were analyzed by ELISA. **(I)** Allogeneic T cells were cocultured with these DCs and incubated with BrdU (10 mM, 24 h) to quantify T cell proliferation by BrdU-ELISA. Knockdown of DC-SIGN blocked the lower T cell proliferative activity induced by ectopic MALAT1. **(J)** Flow cytometry assessment of the number of Tregs (CD4^+^, CD25^+^, and Foxp3^+^) in these cocultures, shown as percentages. **(K)** Knockdown of DC-SIGN blocked the increased Tregs numbers induced by ectopic MALAT1. The data are presented as the mean ± SD from at least three independent experiments.* vs control group, *P* < 0.05; ^#^ vs pMALAT1 group, *P* < 0.05.

Dendritic cell-specific intercellular adhesion molecule-3 grabbing nonintegrin, as an immunoregulatory receptor, has been shown to play important roles in DC immune reactivity as well as T cell activation results through the selective upregulation of IL-10 (10, 31–33). To reveal whether DC-SIGN was involved in the modulating effect of MALAT1 on DC function, we detected its expression in DCs after MALAT1 regulation. The mRNA expression of DC-SIGN was significantly increased in DCs with overexpressed MALAT1 but decreased in those with MALAT1 shRNA compared with control DCs, as assessed by qRT-PCR (Figure 3A). Similarly, by flow cytometry analysis, the DC-SIGN expression in DCs was positively regulated by MALAT1 upregulation, while MALAT1 downregulation counteracted this effect (Figures 3B,C). Therefore, DC-SIGN expression in DCs was regulated by MALAT1.

As stated previously, MALAT1 shaped DCs into a tolerogenic state with a selectively high IL10 secretion. We proposed that ectopic MALAT1 favors tDCs possibly *via* DC-SIGN leading to enhanced IL10. To better understand the role of DC-SIGN in MALAT1-conditioned DCs, DC-SIGN-targeted shRNA was delivered into DCs *via* lentiviral infection before MALAT1 regulation, which was accomplished successfully, as assessed by qRT-PCR (Figure 3D). Then, the DCs were preconditioned with or without DC-SIGN shRNA before MALAT1 overexpression and LPS stimulation. As Figure 2 shows, MALAT1 overexpression conditioned DCs into a tolerogenic phenotype with fewer costimulatory molecules and higher IL10 secretion. However, in DCs pretreated with the pMALAT1 vector plus DC-SIGN shRNA, the number of costimulatory molecules was significantly increased compared with that of DCs treated only with the pMALAT1 vector, with no obvious difference compared to control non-treated DCs (Figure 3E). Thus, knock down of DC-SIGN obviously blocked the MALAT1-induced depression of costimulatory markers. More importantly, DC-SIGN knock down by DC-SIGN shRNA impaired the MALAT1-induced upregulation of IL10 protein and mRNA expression in DCs, as assessed by ELISA and qRT-PCR (Figures 3F,G). This suggests that IL10 upregulation by MALAT1 might be DC-SIGN-dependent. Meanwhile, DC-SIGN knockdown also relieved the depressive effect of MALAT1 on inflammatory cytokine (IL-12, IL-6, and IFN-γ) secretion in the culture supernatant of DCs, shown by ELISA (Figure 3H). In addition, knock down of DC-SIGN blocked the lower T cell proliferative activity induced by ectopic MALAT1 in the MLR assay (Figure 3I). The induction of Tregs numbers by MALAT1 overexpression was also prevented by DC-SIGN shRNA (Figures 3J,K). These data showed that DC-SIGN knockdown abrogated the tolerogenic phenotype of DCs induced by ectopic MALAT1, yielding an immunogenic phenotype and revealing that the effects of MALAT1 overexpression on DC tolerogenic function might be partially mediated by DC-SIGN.

### DC-SIGN^+^ Subsets Induced by Enforced MALAT1 Exert More a Potent Tolerogenic Ability

**Figure 4:**
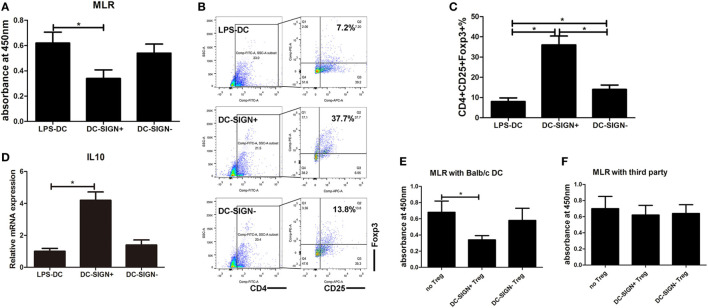
DC-SIGN^+^ subsets induced by enforced MALAT1 exert a more potent tolerogenic ability. After pMALAT1 transfection and LPS stimulation, dendritic cells (DCs) were sorted by MACS into DC-SIGN^+^ DCs and DC-SIGN^−^ DCs. Then, these DCs were conditioned with mitomycin C (10 mg/ml, 2 h) and cocultured with allogeneic T cells for 48 h. **(A)** T cell proliferation initiated by DCs was assessed by BrdU-ELISA. DC-SIGN^+^ DCs exhibited significantly more suppressive effects on primed T cell responses than did LPS-stimulated DCs and DC-SIGN^−^ DCs. **(B,C)** The numbers of Tregs (CD4^+^CD25^+^Foxp3^+^) in T cell cocultures were assessed by flow cytometry **(B)** and are shown as percentages **(C)**. DC-SIGN^+^ subsets, but not DC-SIGN^−^ subsets, induced the generation of Tregs compared with both LPS-DCs and DC-SIGN^−^ DCs. **(D)** IL10 mRNA expression levels in these isolated Tregs were assessed by quantitative real-time reverse transcription PCR. IL10 mRNA levels in Tregs induced by DC-SIGN^+^ populations were significantly higher than those in Tregs induced by LPS-DCs or DC-SIGN^−^ populations. **(E,F)** Tregs (CD4^+^CD25^+^) isolated from different DC and T cell cocultures by MACS were added to a new coculture of mitomycin C-conditioned DCs (syngeneic or third party) and T cells (Treg:T cell:DC ratio of 10:10:1). The suppressive functions of Tregs on DC-primed T cell responses were assessed by BrdU-ELISA. The DC-SIGN^+^ subpopulation-induced Tregs, but not those induced by DC-SIGN^−^ subsets, showed marked inhibition of DC-primed T cell proliferation. The data are presented as the mean ± SD from at least three independent experiments. *P < 0.05; **P < 0.01.

The above studies demonstrated that DC-SIGN is essential for the maintenance of MALAT-conditioned DC tolerogenic functions and that MALAT1 overexpression induced the generation of DC-SIGN^+^ DCs. Therefore, we hypothesized that DC-SIGN^+^ subpopulations of DCs might be the main effector cells in MALAT1 tolerogenic induction. First, DC-SIGN^+^ DCs were sorted by MACS in DCs overexpressing MALAT1 (Figure S3 in Supplementary Material). Unexpectedly, DC-SIGN^+^ DCs exhibited significantly more suppressive effects on primed T cell responses than did LPS-stimulated DCs and DC-SIGN^−^ DCs (Figure 4A). DC-SIGN^+^ subsets, but not DC-SIGN^−^ subsets, induced the generation of Tregs compared with both LPS-DCs and DC-SIGN^−^ DCs (Figures 4B,C). In addition, IL10 mRNA levels in Tregs induced by DC-SIGN^+^ populations were significantly higher than those induced by LPS-DCs or DC-SIGN^−^ populations (Figure 4D). Moreover, DC-SIGN^+^ subpopulation-induced Tregs, but not those induced by DC-SIGN^−^ subsets, showed marked inhibition of DC-primed T cell proliferation, as assessed by MLR (Figures 4E,F). These data demonstrated that DC-SIGN^+^ DC subpopulations induced more Tregs with immunosuppressive effects.

### MALAT1 Promotes DC-SIGN Expression by Functioning as an miR155-5p Sponge in the DC Cytoplasm

**Figure 5:**
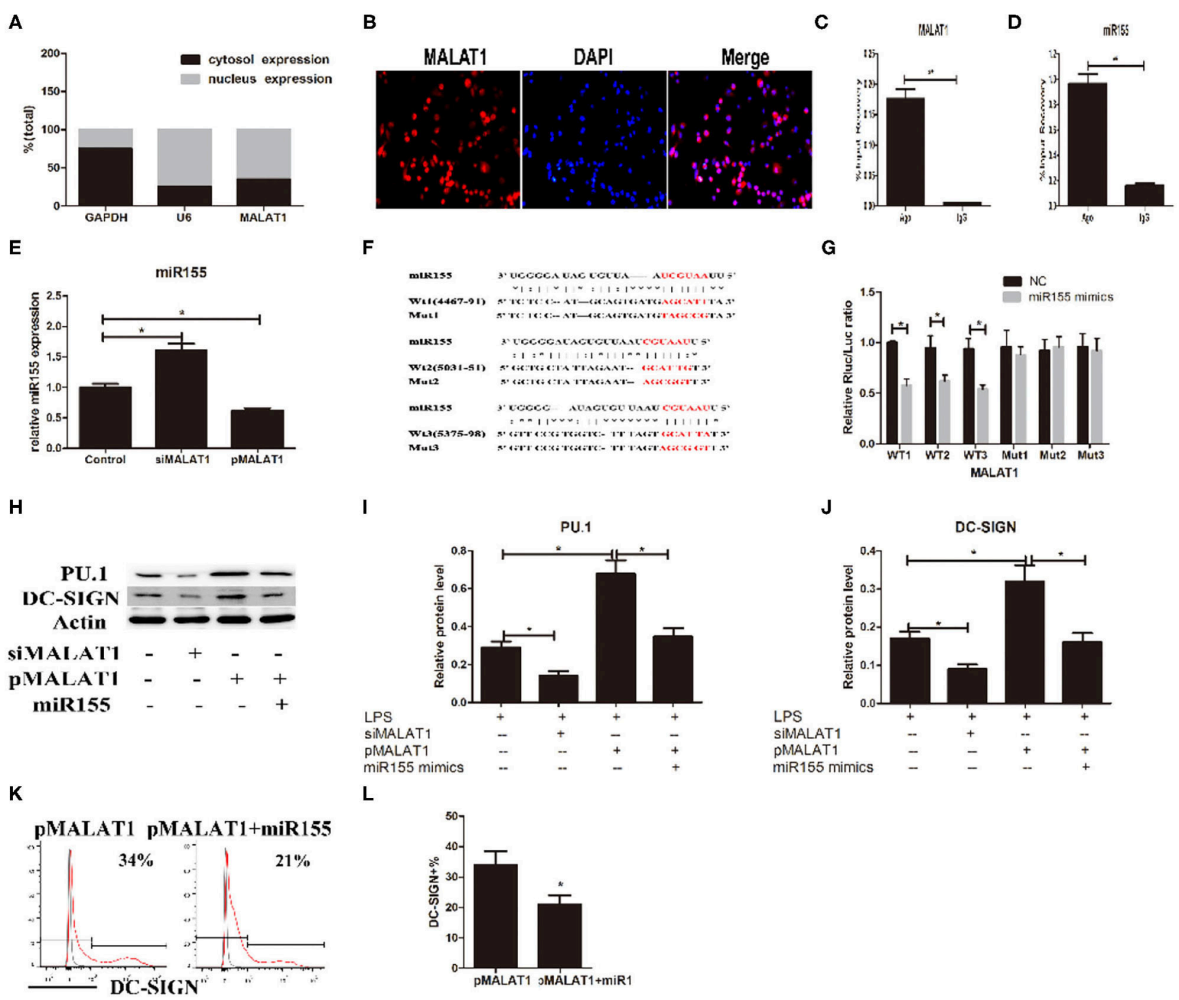
MALAT1 promotes dendritic cell-specific intercellular adhesion molecule-3 grabbing nonintegrin (DC-SIGN) expression by functioning as an miR155-5p sponge. **(A)** MALAT1 expression levels were measured by quantitative real-time reverse transcription PCR (qRT-PCR) in nuclear and cytosolic extracts of LPS-stimulated dendritic cells (DCs). The cytosol and nuclear markers used were GAPDH and U6. **(B)** RNA-FISH was performed to detect MALAT1 expression in LPS-stimulated DCs. Nuclei, blue; MALAT1, red. MALAT1 was in both the nucleus and cytoplasm of DCs. **(C,D)** Total cellular fractions were isolated from LPS-stimulated DCs and immunoprecipitated using Ago2 or IgG antibodies in RNA immunoprecipitation assays. MALAT1 and miR155 levels in the immunoprecipitated complex were detected by qRT-PCR. Significant enrichment of MALAT1 was observed in Ago2 immunoprecipitates compared with that in IgG control immunoprecipitates. miR155 was observed in Ago2 immunoprecipitates and compared with that in IgG control immunoprecipitates. **(E)** The miR155 levels were detected by qRT-PCR in DCs transfected with siMALAT1 or pMALAT1 before LPS stimulation. miR-155 was upregulated in the shMALAT1-treated DCs. **(F)** Schematic illustration of the three miR-155 targeting sites on the MALAT1 gene, WT1(4467–91), WT2(5031–51), and WT3(5375–98). The created mutations (Mut1, Mut2, and Mut3) are shown corresponding to each wild-type (WT), respectively. **(G)** Luciferase reporters containing one of three WT or one of three corresponding mutant putative miR155 binding sites in MALAT1 were constructed. DCs were infected with or without miR-155 mimics and then transfected with luciferase constructs of WT1, WT2, WT3, Mut1, Mut2, or Mut3, respectively. Luciferase activity was analyzed 48 h after transfection. **(H–J)** DCs were transfected with siMALAT1, pMALAT1, or the combination of pMALAT1 and miR155 mimics before LPS stimulation. PU.1 and DC-SIGN protein levels were detected by western blot **(H)**. MALAT1 knockdown significantly decreased PU.1 and DC-SIGN levels in DCs, and MALAT1 overexpression significantly increased PU.1 and DC-SIGN levels compared with those in the control group. Treatment with miR155 mimics partially abolished the effects of MALAT1 on PU.1 and DC-SIGN levels. **(K,L)** DCs were transfected with pMALAT1 or the combination of pMALAT1 and miR155 mimics before LPS stimulation, and DC-SIGN expression levels in DCs were also assessed by flow cytometry **(K)** and shown as percentages **(L)**. The data are presented as the mean ± SD from at least three independent experiments. *P < 0.05; **P < 0.01.

How does MALAT1 regulate the expression of DC-SIGN? Recent evidence has demonstrated that lncRNAs may participate in the ceRNAs regulatory network to regulate a diversity of biological functions. Subcellular localization determination *via* qRT-PCR and FISH demonstrated that MALAT1 was in both the nucleus and the cytoplasm of DCs (Figures 5A,B). We speculated that MALAT1 may function as an miRNA sponge to exert its effects. Thus, RIP was performed in DCs using an Ago2 antibody. Significant enrichment of MALAT1 was observed in Ago2 immunoprecipitates compared with that in IgG control immunoprecipitates (Figure 5C). The MALAT1 sequence was found to contain three putative binding sites for miR155, as screened by starBase. Considering that miRNA-155 could regulate DC-SIGN expression, we supposed that MALAT1 might interact with miR-155, resulting in the modulation of DC-SIGN. Thus, miR155 was also observed in Ago2 immunoprecipitates and compared with that in IgG control immunoprecipitates (Figure 5D). Furthermore, we tested the effects of shMALAT1 and pMALAT1 on the expression of miR-155 in DCs. Notably, miR-155 was upregulated in the shMALAT1-treated DCs (Figure 5E), suggesting that MALAT1 could affect the expression of miR-155. Due to MALAT1 containing three putative binding sites with miR155, a schematic illustration of three miR-155 targeting sites on the MALAT1 gene, WT1(4467–91), WT2(5031–51), and WT3(5375–98), is shown (Figure 5F). The created mutations (Mut1, Mut2, and Mut3) are shown corresponding to each wild-type (WT), respectively (Figure 5F). To validate that MALAT1 was indeed targeted by miR-155, luciferase reporters containing one of three WT or one of three corresponding mutant putative miR155 binding sites on MALAT1 were constructed (Figure 5G). As shown in Figure 5G, the miR155 mimics significantly reduced the luciferase activity of the WT1, WT2, and WT3 MALAT1 reporters. However, the luciferase activity in cells transfected with the MALAT1 mutant 1–3 reporters was not affected by the miR155 mimics. Therefore, by functioning as an miR155 sponge, MALAT1 may affect the target gene expression of miR155. Interestingly, DC-SIGN has been reported to be indirectly targeted by miR155 *via* the direct inhibition of PU.1 (21). MALAT1 knockdown significantly decreased PU.1 and DC-SIGN levels in DCs, and MALAT1 overexpression significantly increased PU.1 and DC-SIGN levels compared with those in the control group (Figures 5H–J). In addition, treatment with miR155 mimics partially abolished the effects of MALAT1 on PU.1 and DC-SIGN levels (Figures 5H–J). The DC-SIGN level was confirmed by flow cytometry (Figures 5K,L). Taken together, these data suggest that MALAT1 may act as an miRNA-155 sponge by inhibiting PU.1 upregulation of DC-SIGN expression in DCs.

### Adoptive Transfer of MALAT1-Overexpressing DCs Protected Mice From Acute Rejection After Cardiac Transplantation and Induced Tregs Expansion

**Figure 6:**
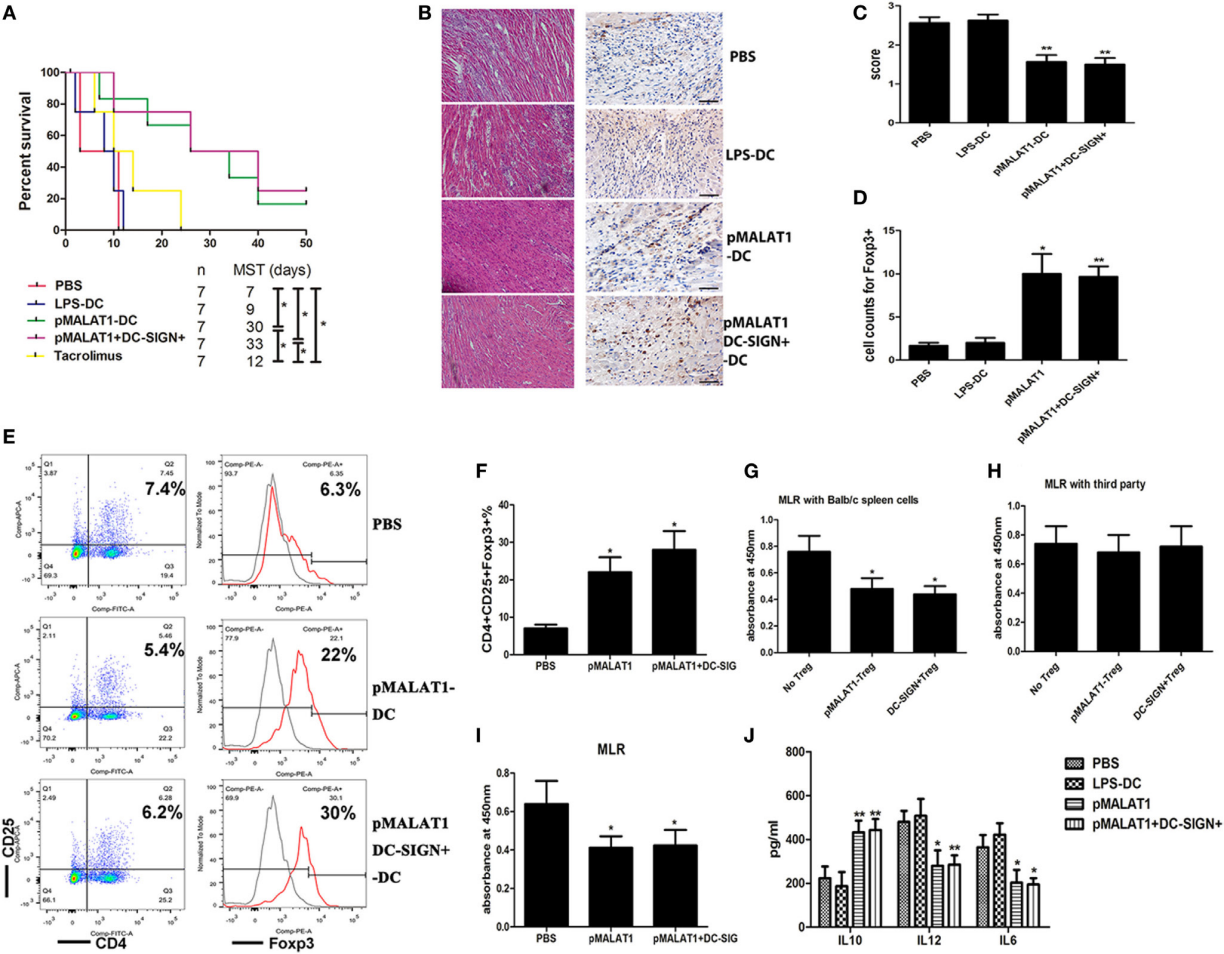
Adoptive transfer of MALAT1-overexpressing dendritic cells (DCs) protected mice from acute transplant rejection and induced Tregs expression. Transplant-recipient mice were transfused with phosphate-buffered saline (PBS), LPS-stimulated DCs, MALAT1-overexpressing DCs (pMALAT1-DCs), or sorted DC-SIGN^+^ cells from MALAT1-overexpressing DCs (pMALAT1^+^DC-SIGN^+^) before transplantation or oral treatment with 1 mg/kg/day of tacrolimus for 7 days. **(A)** Graft survival times of recipients. Abbreviation: MST, median survival time. The survival times in the groups were compared using Mann–Whitney *U* testing. Transfusion with MALAT1-overexpressing DCs or DC-SIGN^+^ DC populations significantly prolonged cardiac allograft survival compared with LPS-DC and PBS transfusion. **(B)** Histologic studies of cardiac allografts harvested 7 days after transplantation were stained with hematoxylin and eosin (H&E) (left) and immunohistochemically stained for Foxp3 (right) [**(B)**, original magnification 20×]. **(C)** Assessment of H&E staining by grading according to the 2005 classification of the International Society for Heart and Lung Transplantation for acute cellular rejection. **(D)** Cell counts of infiltrating Foxp3^+^ cells in cardiac allografts from each group 7 days post-transplantation by immunohistochemical staining. Cardiac allografts from recipients transfused with MALAT1-overexpressing DCs and DC-SIGN^+^ subpopulations had more Foxp3-positive staining cells than did those from recipients transfused with LPS-DC and PBS. The data indicate mean ± SD values derived from five samples in each group and were compared using analysis of variance with the Ryan method. **(E,F)** Tregs (CD4^+^CD25^+^ Foxp3^+^) in splenic T cells were assessed by flow cytometry **(E)** and are shown as percentages **(F)**. Treg numbers were significantly elevated in the spleens of recipient mice injected with pMALAT1-DCs or pMALAT1-DC-SIGN^+^ DCs. Filled histograms represent isotype-matched irrelevant specificity controls. **(G,H)** Tregs (CD4^+^CD25^+^) isolated from recipients transfused with pMALAT1-conditioned DCs by MACS were added to cocultures with mitomycin C-treated splenic cells (from BALB/c mice or third-party mice) and T cells. The suppressive ability of Tregs was assessed by T cell proliferation using BrdU-ELISA. Tregs significantly decreased T cell proliferation when cocultured with donor splenic cells, but not when cocultured with third-party splenic cells. **(I)** Splenic T cells were separated from recipient mice at day 7 post-transplantation. The proliferating activity of splenic T cells was assessed by BrdU-ELISA. In the recipients transfused with pMALAT1-DCs or pMALAT1-DC-SIGN^+^ DCs, splenic T cell proliferation was significantly suppressed. **(J)** IL10, IL12, and IL6 concentrations in the sera of recipient mice were measured by ELISA. The IL12 and IL6 in serum were significantly decreased and IL10 was significantly increased in mice transfused with pMALAT1-DCs or pMALAT1-DC-SIGN^+^ DCs. The data are presented as the mean ± SD from at least five independent experiments. *P < 0.05; **P < 0.01. pMALAT1-DCs, MALAT1-overexpressing DCs; pMALAT1^+^DC-SIGN^+^, DC-SIGN^+^ subsets from MALAT1-overexpressing DCs.

We next used a conditioned DC adoptive transfer strategy to assess the effects of MALAT1-overexpressing DCs on allograft immunity in a murine cardiac transplantation model. Transfusion with MALAT1-overexpressing DCs or DC-SIGN^+^ DC populations significantly prolonged cardiac allograft survival compared with LPS-DC and PBS transfusion (Figure 6A). Histological examination of cardiac allografts showed significantly reduced inflammatory cell infiltration in recipient mice transfused with MALAT-overexpressing DCs or DC-SIGN^+^ DCs compared with that in mice transfused with PBS (Figure 6B), with similar results obtained by grading (Figure 6C). In addition, an immunohistochemical study showed that cardiac allografts from recipients transfused with MALAT1-overexpressing DCs and DC-SIGN^+^ subpopulations had more Foxp3-positive stained cells than those from recipients transfused with LPS-DC and PBS. This reveals that transfusion with MALAT1-overexpressing DCs or DC-SIGN^+^ DCs induced increased Tregs infiltration in cardiac allografts (Figure 6D). Moreover, Treg numbers were significantly elevated in the spleens of recipient mice injected with pMALAT1-DCs or pMALAT1-DC-SIGN^+^ DCs (Figures 6E,F). Furthermore, these Tregs significantly decreased T cell proliferation when cocultured with donor splenic cells but not when cultured with third-party splenic cells (Figures 6G,H), suggesting that MALAT1-overexpressing DC and DC-SIGN^+^ populations induced more antigen-specific Tregs in allografts *in vivo*. Consistently, in the recipients transfused with pMALAT1-DCs or pMALAT1-DC-SIGN^+^ DCs, splenic T cell proliferation was significantly suppressed (Figure 6I), and the inflammatory cytokines IL12 and IL6 in the serum were significantly decreased, although IL10 was also significantly increased (Figure 6J) compared with the responses in the mice transfused with LPS-DCs or PBS. These results suggest that pMALAT1-DC-SIGN^+^ DCs prevented allograft rejection and induced more antigen-specific Tregs *in vivo*.

### *In Vivo* Transfer of MALAT1-Overexpressing DCs Deferred Autoimmune Myocarditis Progression

**Figure 7:**
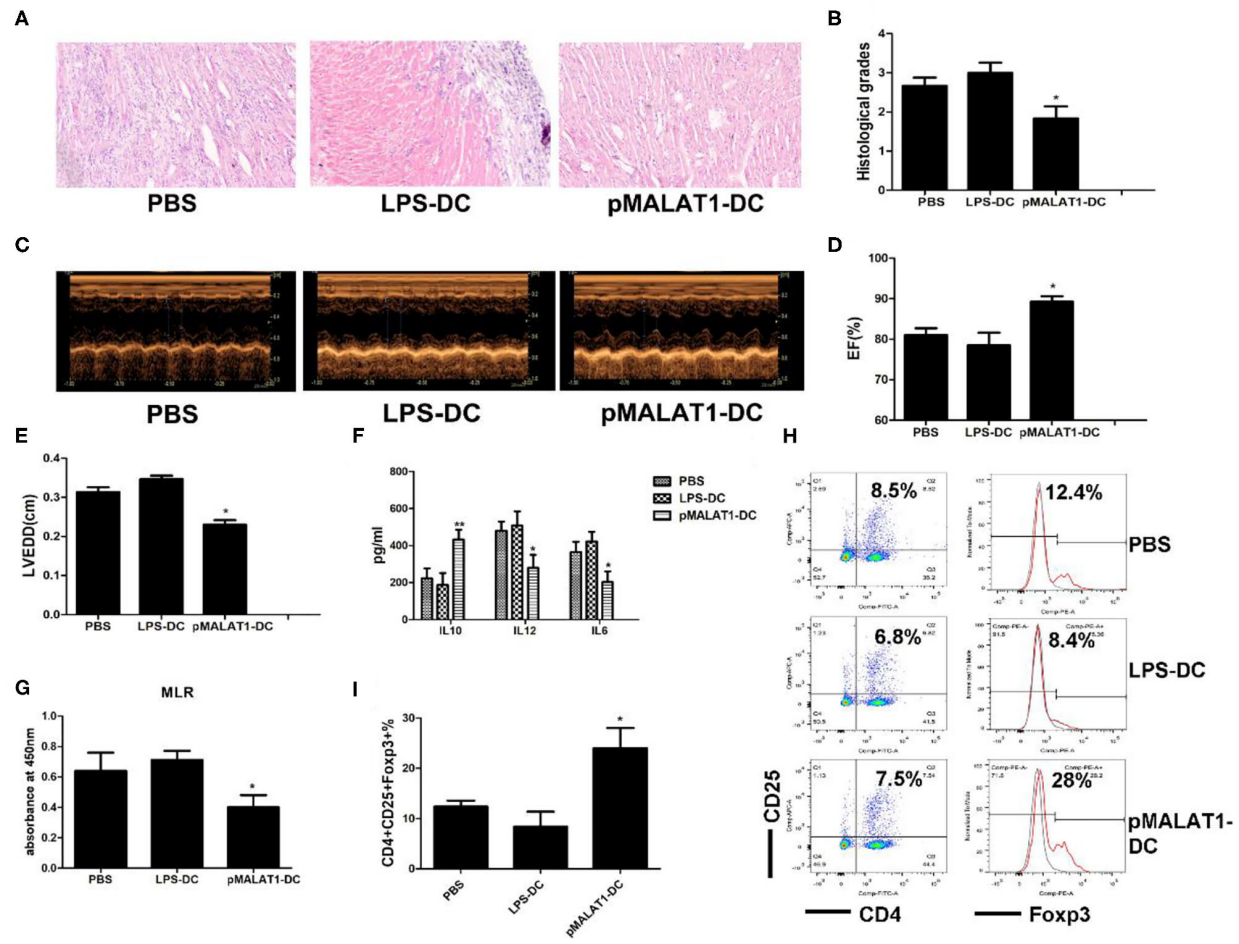
*In vivo* transfer of MALAT1-overexpressing dendritic cells (DCs) deferred autoimmune myocarditis progression. After induction of experimental autoimmune myocarditis (EAM) (immunization with α-myosin H-chain peptide), mice were transfused with MALAT1-overexpressing DCs, LPS-DC, or phosphate-buffered saline (PBS), respectively. Hearts were collected on day 21 post-immunization. **(A)** Consecutive cardiac sections were stained with hematoxylin and eosin (H&E) (original magnification 20×). **(B)** Analysis of H&E staining by grading as described in Section “Materials and Methods.” Transfusion with MALAT1-overexpressing DCs significantly alleviated acute myocardial inflammation in EAM mice compared with PBS transfusion. **(C–E)** Myocardial function was evaluated by echocardiography **(C)** on day 42 post-immunization, and the parameters of LVEDDs **(E)** and EF **(D)** were as shown. Animals injected with MALAT1-overexpressing DCs showed less LV and LVEDDs and more EF than did those that received LPS-DCs and PBS transfusion. **(F–I)** Splenic T cells were separated from EAM mice at day 21 post-immunization. **(F)** IL10, IL12, and IL6 production in the serum of EAM mice was detected by ELISA. The expressions of IL12 and IL6 were significantly decreased and that of IL10 was increased in mice injected with pMALAT1-DCs. **(G)** BrdU-ELISA determined the proliferating activity of splenic T cells. **(H,I)** Tregs (CD4^+^CD25^+^Foxp3^+^) in splenic T cells were assessed by flow cytometry **(H)** and are shown as percentages **(I)**. Filled histograms represent isotype-matched irrelevant specificity controls. The data are presented as the mean ± SD from at least three independent experiments. *P < 0.05; **P < 0.01.

To further investigate the potential effects of MALAT1-overexpressing DCs on autoimmune myocarditis, an adoptive EAM mouse model was induced as described, and the hearts were collected at the peak of disease. Histological examination revealed that transfusion with MALAT1-overexpressing DCs significantly alleviated acute myocardial inflammation in EAM mice compared with PBS transfusion (Figure 7A), and similar results were observed in histological grades (Figure 7B). By contrast, there was no distinct difference in disease severity between mice that received LPS-DCs and PBS transfusion. Animals injected with MALAT1-overexpressing DCs showed lower left ventricular (LV) end-diastolic diameters (LVEDDs) and a higher ejection fraction (EF) than those receiving LPS-DCs and PBS transfusion, as determined by echocardiography (Figures 7C–E). We further evaluated whether the transfer of pMALAT1-DCs was able to promote the secretion of cytokines and induce T cell hyporesponsiveness and found that the expression of inflammatory cytokines IL12 and IL6 in serum was significantly decreased, and the anti-inflammatory cytokine IL10 was increased in mice injected with pMALAT1-DCs (Figure 7F) but not in those transfused with LPS-DCs or PBS. As expected, mice that received pMALAT1-DCs showed significantly suppressed T cell proliferative potential (Figure 7G) and increased Treg numbers in the spleen (Figures 7H,I) compared with those injected with LPS-DCs and PBS.

## Discussion

Dendritic cells, as the most potent antigen-presenting cells, control the outcome of innate and adaptive immune responses through phenotype and functional switching. tDCs, which tolerize T cell immunity and induce Tregs, are considered attractive targets in therapeutic approaches aiming to establish immune tolerance after transplantation or in autoimmune diseases (4, 5, 7). Some transcription factors, anti-inflammatory cytokines, and miRNAs have been reported to play roles in the modulation of DC function; however, the role of lncRNAs in DC function is not well understood. Only the lncRNAs lnc-DC and HOTAIRM1 have been shown to play a regulatory role in human DC differentiation (34, 35). Our study first revealed that the functional lncRNA MALAT1 is involved in the functional modulation of murine BMDCs and further confirmed its regulation of tolerance through the switching of DC functions.

The MALAT1 lncRNA is highly conserved among mammals and was initially discovered with established roles in lung cancer (27, 36). MALAT1 has also been reported to be involved in endothelial cell function and cardiovascular disease (37, 38). Recent publications have assigned immune regulatory functions to MALAT1 (28, 39, 40). Specifically, MALAT1 plays a role in innate immune responses by depressing NF-κB activity in macrophages (28). Moreover, MALAT1-associated small cytoplasmic (masc) RNA has been shown to play a role in cardiovascular innate immunity (41). By contrast, in activated DCs, MALAT1 plays a tolerizing or regulatory role in DC function in a cell type-specific manner. Ectopic MALAT1 favors DCs in a tolerogenic state and induces a more potent tolerogenic subset: the DC-SIGN^+^ DC population. Moreover, in the *in vitro* study, the MALAT1-overexpressing DCs showed fewer costimulatory molecules and selective high IL10 secretion, resulting in more Tregs with antigen-specific suppression. To further confirm the tolerogenic function of MALAT1-overexpressing DCs *in vivo*, we transferred these DCs into the mice models of heart transplantation or EAM. The *in vivo* study revealed that the adoptive transfusion of MALAT1-conditioned DCs and DC-SIGN^+^ subsets prevented allograft rejection. In addition to its role in transplantation, the transfer of MALAT1 enforced the protection of DCs from the development of EAM in mice, further proving its immune-tolerant effect in autoimmune disease.

How can these conditioned DCs induce tolerance *in vivo* in the specific models? As a therapeutic strategy, tDC therapy has been demonstrated to be a promising novel immunotherapeutic tool in immune-related diseases, like transplantation, autoimmunity, allergy, and cancer (2, 42–45). The therapeutic effects of tDCs on experimental animal models have been verified. In transplantation, the transfer of different conditioned tDCs has been found to extensively prolong allograft survival (5, 46, 47). There have also been numerous reports of prevention or relief of autoimmune disease following conditioned tDCs infusion (2, 43). It is likely that TolDC will be a novel and innovative immunotherapy in the future. For effective conditioned tDCs therapy, the mechanism of action is likely to be critical. The reported mechanisms by which tDCs induce immune tolerance *in vivo* include T cell deletion, induction of T cell hyporesponsiveness, modulation of the T cell cytokine profile, induction of antigen-specific Tregs, and depression of immunosuppressive molecules (45, 48, 49). The main advantage of tDCs therapy is the induction of antigen-specific Tregs *in vivo*, which provides the advantage of antigen specificity rather than overall immune suppression (48, 50–52). The DCs with expanded antigen-specific Tregs exert effective antigen-specific suppression in MLRs and block immune response *in vivo*. Moreover, these DCs with expanded Tregs might regulate ongoing immune responses and provide long-lasting therapeutic effects (50, 52, 53). Therefore, the expansion of Tregs from conditioned DCs provides a promising method for antigen-specific control of unwanted immune responses. In our study, we conducted two *in vivo* experiments to investigate the function of MALAT1-conditioned DCs *in vivo*. In one, the transfusion of MALAT1-overexpressing DCs and the DC-SIGN^+^ subset in heart transplantation not only induced immune tolerance but also induced infiltration of more Foxp3^+^ Tregs and more splenic Tregs with antigen-specific immunosuppressive effects into allografts. In the second experiment using an EAM mice model, the transfusion of MALAT1-overexpressing DCs also induced an increase in the splenic Tregs number. Therefore, MALAT1-conditioned DCs induce immune tolerance *in vivo*, possibly partially resulting from the antigen-specific Tregs induction. However, whether the effectiveness of the transferred DCs *in vivo* is dose dependent or dependent on the cytokine concentration, like IL10, and, thereby, antigen-aspecific is still in need of further study to confirm.

As a cell receptor, DC-SIGN mediates the functions of DCs and macrophages in presenting and shaping T cell immunity (12, 13). It is involved in immunosuppressive maintenance after transplantation and during tumor growth and pathogenic infection (11, 14, 15). In particular, miR155 has been found to be a prominent regulator of innate and adaptive immune responses (17, 19) because it modulates DC maturation and function as well as allograft rejection after transplantation (18, 20, 54, 55). In this study, we found that ectopic MALAT1 promoted DC-SIGN expression by functioning as an miR155 sponge in the cytoplasm, which is essential for the tolerogenic maintenance of DCs. Surprisingly, among these cells, the DC-SIGN-positive subset exhibited a more potent tolerogenic ability. This result was further supported by the upregulation of DC-SIGN expression in tolerized cardiac allografts detected in our microarray data. A previous study found that DC-SIGN promotes the secretion of IL10 by macrophages after transplantation (15, 56). The anti-inflammatory cytokine IL10 plays regulatory roles in immune-related diseases, particularly in transplantation and autoimmune-related diseases (15, 29). In this study, we found that IL10 is also required for the DC-SIGN-mediated tolerogenic functions of DCs after MALAT1 overexpression. Therefore, overexpressed MALAT1 induces tDCs and immune tolerance in transplantation and autoimmune disease by the miR155/DC-SIGH/IL10 axis.

With respect to organ transplantation, only a few studies have investigated lncRNAs and transplant immunity. lncRNAs have been studied as biomarkers for transplant rejection in renal transplantation recipients (26, 57). One published study that investigated cardiac transplantation revealed the profile of lncRNAs in cardiac allograft rejection and suggested that two lncRNAs, namely, lncRNA-A930015D03Rik and mouselincRNA1055, are involved in allograft rejection by regulating the Th1 cell response (26). lncRNAs have been identified as crucial modulators of autoimmunity process and are involved in regulating the pathogenesis of autoimmune diseases (58–60). A current study has indicated that downregulated lnc-Smad3 exerts a protective effect on colitis by recruiting Ash1l to the Smad3 promoter and regulating Tregs polarization (61). In addition, one study demonstrated that an inflammatory regulator of lncRNA-NEAT1 was positively linked to systemic lupus erythematosus activity (62). However, the tolerogenic role of lncRNAs in both transplant rejection and autoimmune disease remains poorly understood. In particular, lncRNA-based tDCs immunotherapy in cardiac graft rejection and autoimmune myocarditis has not been reported prior to our study. We first revealed the profiles of differentially expressed lncRNAs and identified MALAT1 lncRNA in tolerized cardiac allografts and infiltrating cells, further supporting the hypothesis that MALAT1 is involved in allograft tolerance induction by modulating DCs function *in vivo*. Finally, we successfully validated the therapeutic effect of MALAT1 on cardiac graft rejection and autoimmune myocarditis by the transfer of MALAT1-overexpressing DCs. These results suggest that lncRNA serves as a novel tolerance regulator in therapeutic intervention for organ transplantation and autoimmunity diseases. Nevertheless, the precise mechanism of MALAT1 lncRNA in immune tolerance induction should be characterized in the future *in vivo*.

In conclusion, we first found that the functional lncRNA MALAT1 is involved in the induction of tDCs and immune tolerance in heart transplantation and EAM. MALAT1 exerts a novel regulatory role in promoting the tolerogenic function of DCs and the induction of immune tolerance *in vitro* and *in vivo*. Mechanistically, MALAT1 promotes DC-SIGN and IL10 production by functioning as an miR155 sponge in the cytoplasm, which is essential for the induction and maintenance of tDCs and for the more potent tolerogenic function of the DC-SIGN-positive subsets. This study provides a comprehensive functional and mechanistic characterization of MALAT1 with respect to tDCs and immune tolerance. Therapeutically, MALAT1 lncRNA is a novel tolerance regulator with important implications in settings involving DCs, such as transplantation, autoimmune diseases, cancer, and pathogenic infection.

## Ethics Statement

All experimental protocols were approved by the Institutional Animal Care and Use Committee at Harbin Medical University. This study was conducted in accordance with the Guide for the Care and Use of Laboratory Animals (Institute of Laboratory Animal Resources/National Institutes of Health, Bethesda, MD, USA).

## Author Contributions

Conceptualization: JW and MZ. Methodology, validation, and formal analysis: HZ and ML. Investigation: YZ, XJ, QZ, SZ, JT, MS, and XL. Writing—original draft: JW and YS. Writing—reviewing and editing: BY and SL. Visualization: YW and MZ. Supervision: MZ. Project administration: JW and MZ. Funding acquisition, JW and MZ.

## Conflict of Interest Statement

The authors declare that the research was conducted in the absence of any commercial or financial relationships that could be construed as a potential conflict of interest.

## Supplementary Material

Figure S1 Long noncoding RNA (lncRNA) MALAT1 is upregulated in tolerized cardiac allografts. **(A)** lncRNA microarray analysis was performed with RNA isolated from tolerized and rejected cardiac allografts in mice at day 7 post-transplantation. The heat map shows 50 differentially expressed lncRNAs (≥1.5-fold change) in tolerized cardiac allografts vs. rejected allografts. **(B)** The lncRNA MALAT1 in the allografts was selectively confirmed by quantitative real-time reverse transcription PCR (qRT-PCR) (*n* = 5). **(C)** MALAT1 expression was detected by qRT-PCR in graft-infiltrating cells isolated from cardiac allografts (*n* = 5). All values of lncRNA expression levels were normalized to β-actin, **P* < 0.05. **(D)** RNA-FISH analysis was performed with a MALAT1 probe to detect the expression of MALAT1, and immunostaining with CD11c antibodies also performed to detect the dendritic cells (DCs) location in tolerized cardiac allografts. MALAT1 were labeled with rhodamine (red). CD11c were labeled with FAM (green). Nuclei were labeled with DAPI (blue).

Figure S2 Gating strategy for the phenotypic detection of Treg cell: lymphocytes → doublet exclusion (FSC and SSC) → CD4^+^ T cells → CD25^+^Foxp3^+^ T cells. Abbreviations: FSC, forward scatter; SSC, side scatter; A, area; H, height; W, width.

Figure S3 Example of the separation of DC-SIGN^+^ populations from MALAT1-overexpressing DCs by MACS. The purities of DC-SIGN^+^ DC populations were determined by FACS analysis.

## Abbreviations

DCs, dendritic cells; DC-SIGN, dendritic cell-specific intercellular adhesion molecule-3 grabbing nonintegrin; EAM, experimental autoimmune myocarditis; lncRNAs, long noncoding RNAs; tDC, tolerogenic dendritic cell.
