# Corrigendum: The Long Noncoding RNA MALAT1 Induces Tolerogenic Dendritic Cells and Regulatory T Cells *via* miR155/Dendritic Cell-Specific Intercellular Adhesion Molecule-3 Grabbing Nonintegrin/IL10 Axis

**DOI:** 10.3389/fimmu.2020.582491

**Published:** 2020-10-16

**Authors:** Jian Wu, Hanlu Zhang, Yang Zheng, Xiangyuan Jin, Mingyang Liu, Shuang Li, Qi Zhao, Xianglan Liu, Yongshun Wang, Ming Shi, Shengnan Zhang, Jinwei Tian, Yong Sun, Maomao Zhang, Bo Yu

**Affiliations:** ^1^Department of Cardiology, The Second Affiliated Hospital of Harbin Medical University, Harbin, China; ^2^The Key Laboratory of Myocardial Ischemia, Harbin Medical University, Ministry of Education, Harbin, China; ^3^Department of Thoracic Surgery, The Third Affiliated Hospital of Harbin Medical University, Harbin, China; ^4^School of Biomedical Sciences, The University of Hong Kong, Pokfulam, China; ^5^School of Life Science and Technology, Harbin Institute of Technology, Harbin, China

**Keywords:** MALAT1 long noncoding RNA, tolerogenic dendritic cell, immune tolerance, dendritic cell-specific intercellular adhesion molecule-3 grabbing nonintegrin, miR155, IL10

In the original article, there was a mistake in Figure 5A and Figure 7A as published. In the published version of Figure 5A, the group of U6 and GAPHD were interchanged. In addition, in Figure 7A, the H&E staining for the “LPS-DC” group was mistakenly presented with incorrected images. The corrected Figure 5 and Figure 7 appears below.

**Figure 5:**
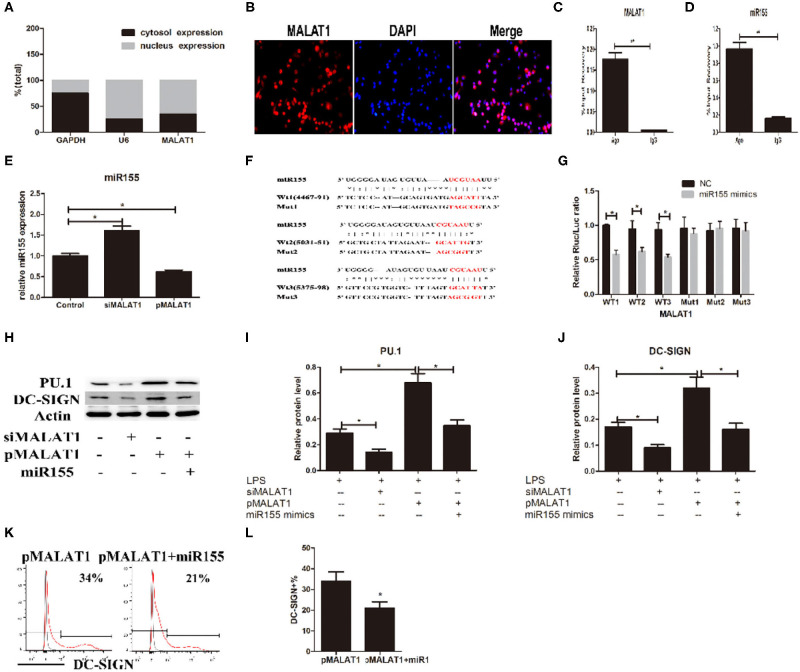
MALAT1 promotes dendritic cell-specific intercellular adhesion molecule-3 grabbing nonintegrin (DC-SIGN) expression by functioning as an miR155-5p sponge. **(A)** MALAT1 expression levels were measured by quantitative real-time reverse transcription PCR (qRT-PCR) in nuclear and cytosolic extracts of LPS-stimulated dendritic cells (DCs). The cytosol and nuclear markers used were GAPDH and U6. **(B)** RNA-FISH was performed to detect MALAT1 expression in LPS-stimulated DCs. Nuclei, blue; MALAT1, red. MALAT1 was in both the nucleus and cytoplasm of DCs. **(C,D)** Total cellular fractions were isolated from LPS-stimulated DCs and immunoprecipitated using Ago2 or IgG antibodies in RNA immunoprecipitation assays. MALAT1 and miR155 levels in the immunoprecipitated complex were detected by qRT-PCR. Significant enrichment of MALAT1 was observed in Ago2 immunoprecipitates compared with that in IgG control immunoprecipitates. miR155 was observed in Ago2 immunoprecipitates and compared with that in IgG control immunoprecipitates. **(E)** The miR155 levels were detected by qRT-PCR in DCs transfected with siMALAT1 or pMALAT1 before LPS stimulation. miR-155 was upregulated in the shMALAT1-treated DCs. **(F)** Schematic illustration of the three miR-155 targeting sites on the MALAT1 gene, WT1(4467–91), WT2(5031–51), and WT3(5375–98). The created mutations (Mut1, Mut2, and Mut3) are shown corresponding to each wild-type (WT), respectively. **(G)** Luciferase reporters containing one of three WT or one of three corresponding mutant putative miR155 binding sites in MALAT1 were constructed. DCs were infected with or without miR-155 mimics and then transfected with luciferase constructs of WT1, WT2, WT3, Mut1, Mut2, or Mut3, respectively. Luciferase activity was analyzed 48 h after transfection. **(H–J)** DCs were transfected with siMALAT1, pMALAT1, or the combination of pMALAT1 and miR155 mimics before LPS stimulation. PU.1 and DC-SIGN protein levels were detected by western blot **(H)**. MALAT1 knockdown significantly decreased PU.1 and DC-SIGN levels in DCs, and MALAT1 overexpression significantly increased PU.1 and DC-SIGN levels compared with those in the control group. Treatment with miR155 mimics partially abolished the effects of MALAT1 on PU.1 and DC-SIGN levels. **(K,L)** DCs were transfected with pMALAT1 or the combination of pMALAT1 and miR155 mimics before LPS stimulation, and DC-SIGN expression levels in DCs were also assessed by flow cytometry **(K)** and shown as percentages **(L)**. The data are presented as the mean ± SD from at least three independent experiments. *P < 0.05; **P < 0.01.

**Figure 7:**
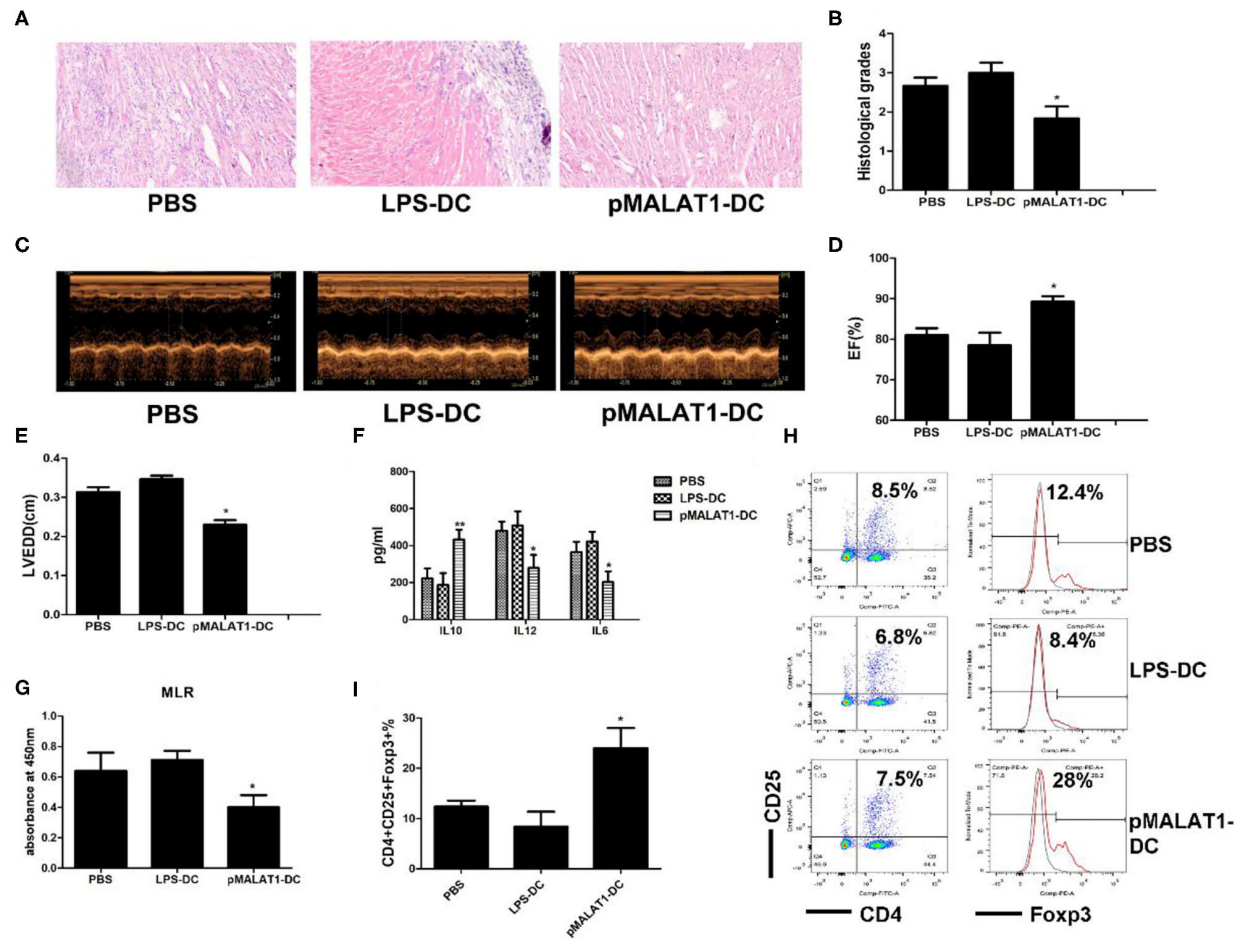
*In vivo* transfer of MALAT1-overexpressing dendritic cells (DCs) deferred autoimmune myocarditis progression. After induction of experimental autoimmune myocarditis (EAM) (immunization with α-myosin H-chain peptide), mice were transfused with MALAT1-overexpressing DCs, LPS-DC, or phosphate-buffered saline (PBS), respectively. Hearts were collected on day 21 post-immunization. **(A)** Consecutive cardiac sections were stained with hematoxylin and eosin (H&E) (original magnification 20×). **(B)** Analysis of H&E staining by grading as described in Section “Materials and Methods.” Transfusion with MALAT1-overexpressing DCs significantly alleviated acute myocardial inflammation in EAM mice compared with PBS transfusion. **(C–E)** Myocardial function was evaluated by echocardiography **(C)** on day 42 post-immunization, and the parameters of LVEDDs **(E)** and EF **(D)** were as shown. Animals injected with MALAT1-overexpressing DCs showed less LV and LVEDDs and more EF than did those that received LPS-DCs and PBS transfusion. **(F–I)** Splenic T cells were separated from EAM mice at day 21 post-immunization. **(F)** IL10, IL12, and IL6 production in the serum of EAM mice was detected by ELISA. The expressions of IL12 and IL6 were significantly decreased and that of IL10 was increased in mice injected with pMALAT1-DCs. **(G)** BrdU-ELISA determined the proliferating activity of splenic T cells. **(H,I)** Tregs (CD4^+^CD25^+^Foxp3^+^) in splenic T cells were assessed by flow cytometry **(H)** and are shown as percentages **(I)**. Filled histograms represent isotype-matched irrelevant specificity controls. The data are presented as the mean ± SD from at least three independent experiments. *P < 0.05; **P < 0.01.

The authors apologize for this error and state that this does not change the scientific conclusions of the article in any way. The original article has been updated.

