# Supplementary figures and images for: The Long Noncoding RNA MALAT1 Induces Tolerogenic Dendritic Cells and Regulatory T Cells via miR155/Dendritic Cell-Specific Intercellular Adhesion Molecule-3 Grabbing Nonintegrin/IL10 Axis
Source: Front Immunol. 2018 Aug 13;9:1847. doi: 10.3389/fimmu.2018.01847 (PMC6099154; doi:10.3389/fimmu.2018.01847)

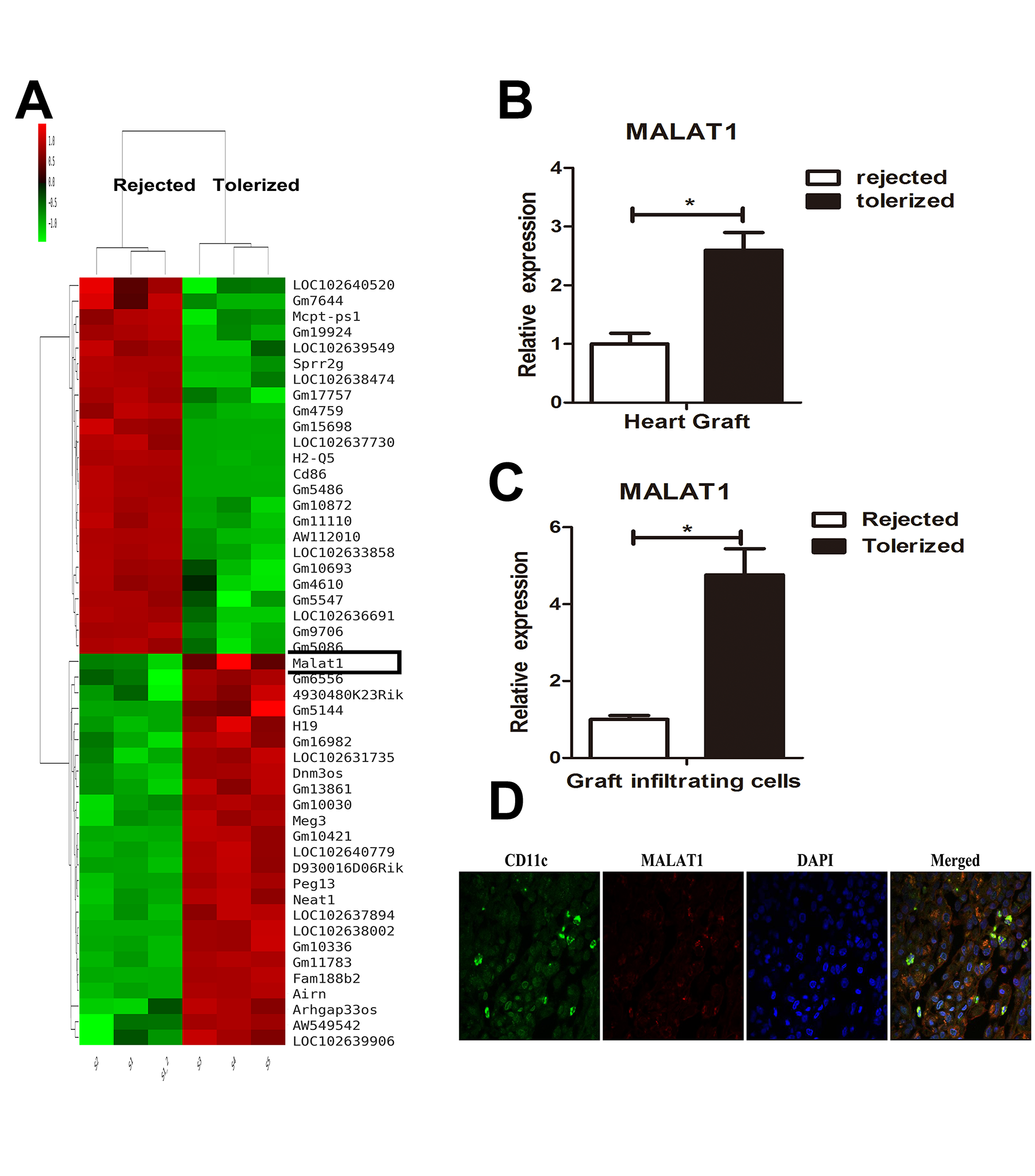

Supplement: Figure S1 — Long noncoding RNA (lncRNA) MALAT1 is upregulated in tolerized cardiac allografts. (A) lncRNA microarray analysis was performed with RNA isolated from tolerized and rejected cardiac allografts in mice at day 7 post-transplantation. The heat map shows 50 differentially expressed lncRNAs (≥1.5-fold change) in tolerized cardiac allografts vs. rejected allografts. (B) The lncRNA MALAT1 in the allografts was selectively confirmed by quantitative real-time reverse transcription PCR (qRT-PCR) (n = 5). (C) MALAT1 expression was detected by qRT-PCR in graft-infiltrating cells isolated from cardiac allografts (n = 5). All values of lncRNA expression levels were normalized to β-actin, *P < 0.05. (D) RNA-FISH analysis was performed with a MALAT1 probe to detect the expression of MALAT1, and immunostaining with CD11c antibodies also performed to detect the dendritic cells (DCs) location in tolerized cardiac allografts. MALAT1 were labeled with rhodamine (red). CD11c were labeled with FAM (green). Nuclei were labeled with DAPI (blue). [file image_1.tif]

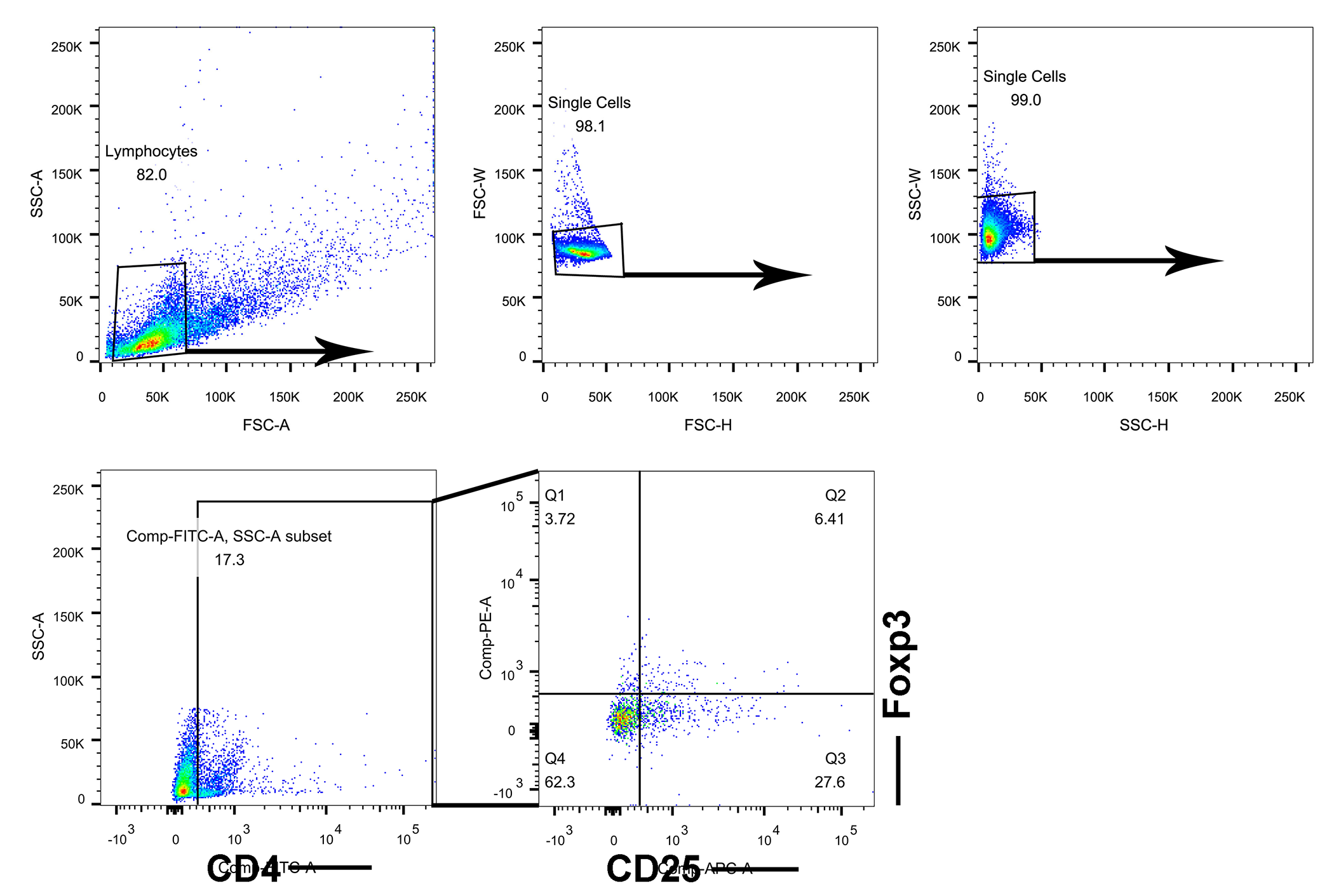

Supplement: Figure S2 — Gating strategy for the phenotypic detection of Treg cell: lymphocytes → doublet exclusion (FSC and SSC) → CD4+ T cells → CD25+Foxp3+ T cells. Abbreviations: FSC, forward scatter; SSC, side scatter; A, area; H, height; W, width. [file image_2.tif]

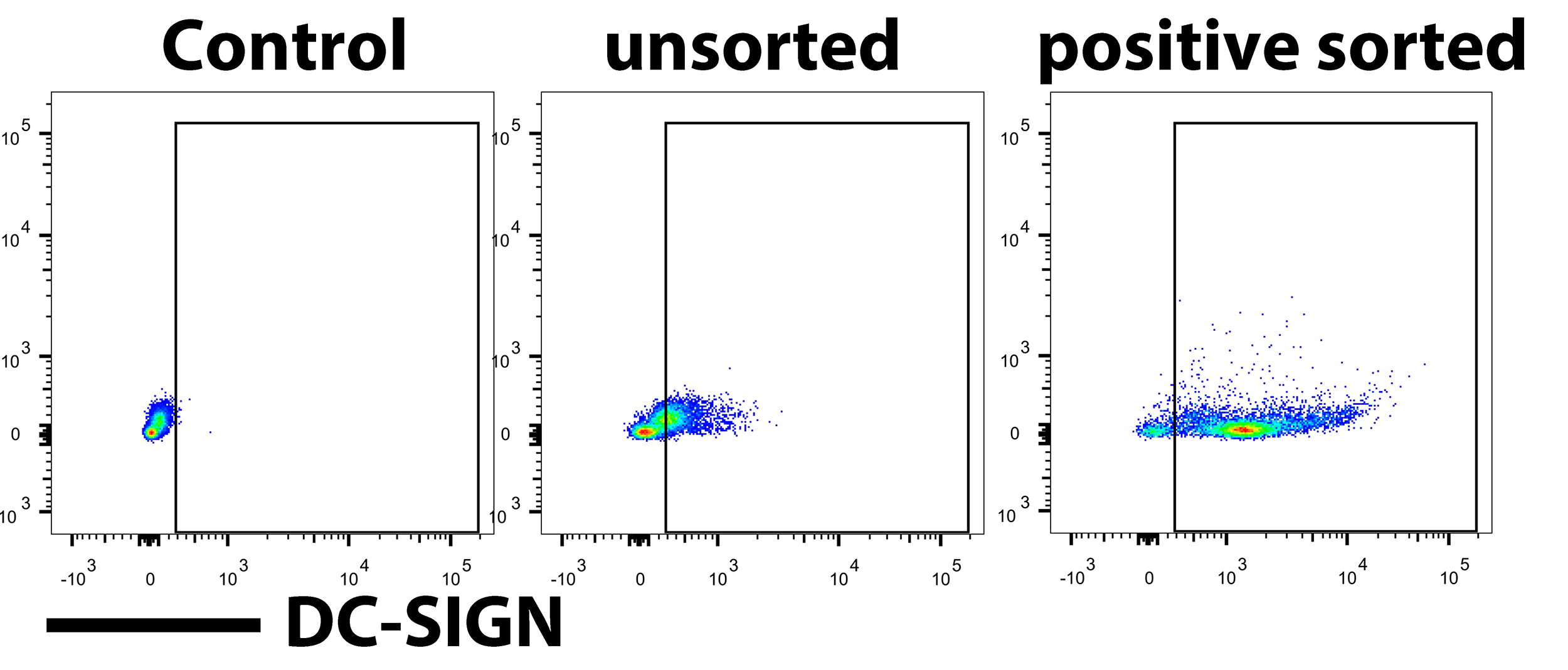

Supplement: Figure S3 — Example of the separation of DC-SIGN+ populations from MALAT1-overexpressing DCs by MACS. The purities of DC-SIGN+ DC populations were determined by FACS analysis. [file image_3.tif]
