# Supplementary material for: The Long Noncoding RNA MALAT1 Induces Tolerogenic Dendritic Cells and Regulatory T Cells via miR155/Dendritic Cell-Specific Intercellular Adhesion Molecule-3 Grabbing Nonintegrin/IL10 Axis
Source: Front Immunol. 2018 Aug 13;9:1847. doi: 10.3389/fimmu.2018.01847 (PMC6099154; doi:10.3389/fimmu.2018.01847)
Supplement: Supplementary file 4 [file table_1.docx]

Supplemental Table 1. Primers used for PCR, ChIP and RIP assays in this study.

| **Primers** | **Sequences** |
| --- | --- |
| **PCR primers** |  |
| MALAT1 | FORWARD: TGAGGACAACAGGTGAACGA  REVERSE: CCCAAGGCCAACATTACATC |
| β-actin | FORWARD: ACGGCCAGGTCATCACTATTG  REVERSE: CAAGAAGGAAGGCTGGAAAAGA |
| IL10 | FORWARD: TAGAGCTGCGGACTGCCTTC  REVERSE: TTCCGATAAGGCTTGGCAAC |
| DC-SIGN | FORWARD: CTGGGAGAGGAAGACTGTGC  REVERSE: ATCACTTGCTAGGGCAGGAA |
| miR155 | FORWARD: GTGATAGGGGTTTTGGCCTCTGA  REVERSE: ATCCAGTGCAGGGTCCGAGG |
| U6 | FORWARD: GCCAGCACCATGCTCTTCTA  REVERSE: GGTTCCACAGATGCTCAGGTC |
| **ChIP primers** |  |
| MALAT1 promoter  Site 1 | FORWARD: CTAAGATCTGGATCTAACCCTTTGGG  REVERSE: GAGATATGAACAGAGGTTGGGGC |
| MALAT1 promoter  Site 2 | FORWARD: CCTGCTCTCTTATAGAACTGAACCAAG  REVERSE: AAGAGATCCTCCACGAGATCCTG |
| GAPDH promoter | FORWARD: CATGGGTGTGAACCATGAGA  REVERSE: GTCTTCTGGGTGGCAGTGAT |
| IL8 | FORWARD: GGGCCATCAGTTGCAAATC  REVERSE: TTCCTTCCGGTGGTTTCTTC |
| **RIP primers** |  |
| MALAT1 | FORWARD: AGGCATTCAGGCAGCGAG  REVERSE: CTTAAGTTTAGAGTTCTAATTCTTTTTACTGCTCA |
| miR155-5p | FORWARD: TTAATGCTAATTGTGATAGGGGT  REVERSE: CTCTACAGCTATATTGCCAGCC |
| U1 (control) | FORWARD: GGGAGATACCATGATCACGAAGGT  REVERSE: CCACAAATTATGCAGTCGAGTTTCCC |
